# Unveiling the immoral ramp feature of dissolved oxygen signals with dynamical systems analysis: perspectives for robust soft-sensor development

**DOI:** 10.1038/s41598-026-43885-y

**Published:** 2026-04-01

**Authors:** Mariane Y. Schneider, Elena Torfs, Juan Pablo Carbajal

**Affiliations:** 1 BIOMATH, Department of Data Analysis and Mathematical Modelling, Ghent University, Coupure Links 653, 9000 Ghent, Belgium; 2 CAPTURE, Centre for Advanced Process Technology for Urban REsource Recovery, Ghent University, Frieda Saeysstraat 1, Ghent, Belgium; 3 DTU Sustain, DTU, Bygningstorvet, building 115, 2800 Lyngby, Denmark; 4 modelEAU, Département de Génie Civil et de Génie des Eaux, Université Laval, Pavillon Adrien-Pouliot, 1065, Av. de la Médecine, Quebec, G1V 0A6 QC Canada; 5 Institute for Energy Technology, OST Eastern Switzerland University of Applied Sciences, Oberseestrasse 10, Rapperswil, Switzerland

**Keywords:** Engineering, Climate change, Environmental monitoring

## Abstract

The water sector is increasingly relying on data-driven approaches to create proxy measurements. These approaches are often trained with data covering only few situations, resulting in a lack of robustness and increasing the risk of false predictions. Therefore, robust approaches are needed for data-driven proxy measurements (i.e. soft sensors), especially for water recovery and reuse. In climate science and robotics, Dynamical Systems Analysis (DSA) is used to explore a wide range of system behaviour and uncover conditions of high uncertainty, and potential tipping points. DSA allows the systematic analysis of model dynamics, and the measurable features caused by these dynamics. We created a novel DSA workflow for soft-sensor development to measure water quality. Herein, we demonstrate that the integration of DSA into soft-sensor development adds robustness by revealing all mathematically possible combinations of state variables that lead to a feature. It can thus detect possible interferences and help design the soft sensor to avoid these. We used DSA for the falsification of a ramp-feature-based soft sensor and found that, despite a published, successful laboratory and real-world application, the ramp feature is immoral. Such an immorality implies that false predictions could occur. However, the DSA analysis uncovered under which operational conditions the ramp becomes a robust soft -sensor feature. Targeted experiments will be necessary to confirm the boundary of the robust conditions in the real world.

## Introduction

The impact of climate change on water cycles has a cascading effect on urban areas, agriculture, industry, and the natural environment, necessitating adaptation. A flexible, circular on-site infrastructure^1–3^, is a promising adaptation strategy. Furthermore, on-site plants may allow cost savings by removing the need for sewer infrastructure^4^.

Monitoring and control pose decisive challenges inherent to on-site water and wastewater infrastructure. This can be attributed, on the one hand, to the high capital cost required to purchase physical water quality sensors. One way to reduce these costs is developing soft sensors^5^ based on inexpensive proxy sensors instead. On the other hand, monitoring is hampered by required sensor maintenance^6^. Most studies do not address the maintenance challenge even for on-site settings (e.g.^7,8^). However, sensors expose a variety of often non-linear wearing effects such as damage, fouling, or deterioration (e.g.^9–12^) and sensor faults that impact the treatment performance^13^. The wearing effects are especially challenging for data-driven approaches, despite accurate results (e.g.^14^). The reason is that wearing effects cause measurements that can lie outside the training dataset. Consequently, sensors should either be installed in an environment where nearly no maintenance is required, for example, a chlorination tank^15^ or features for monitoring^16^ and control^17^ need to be robust to sensor wearing effects such as drift or noise by design.

**Fig. 1:**
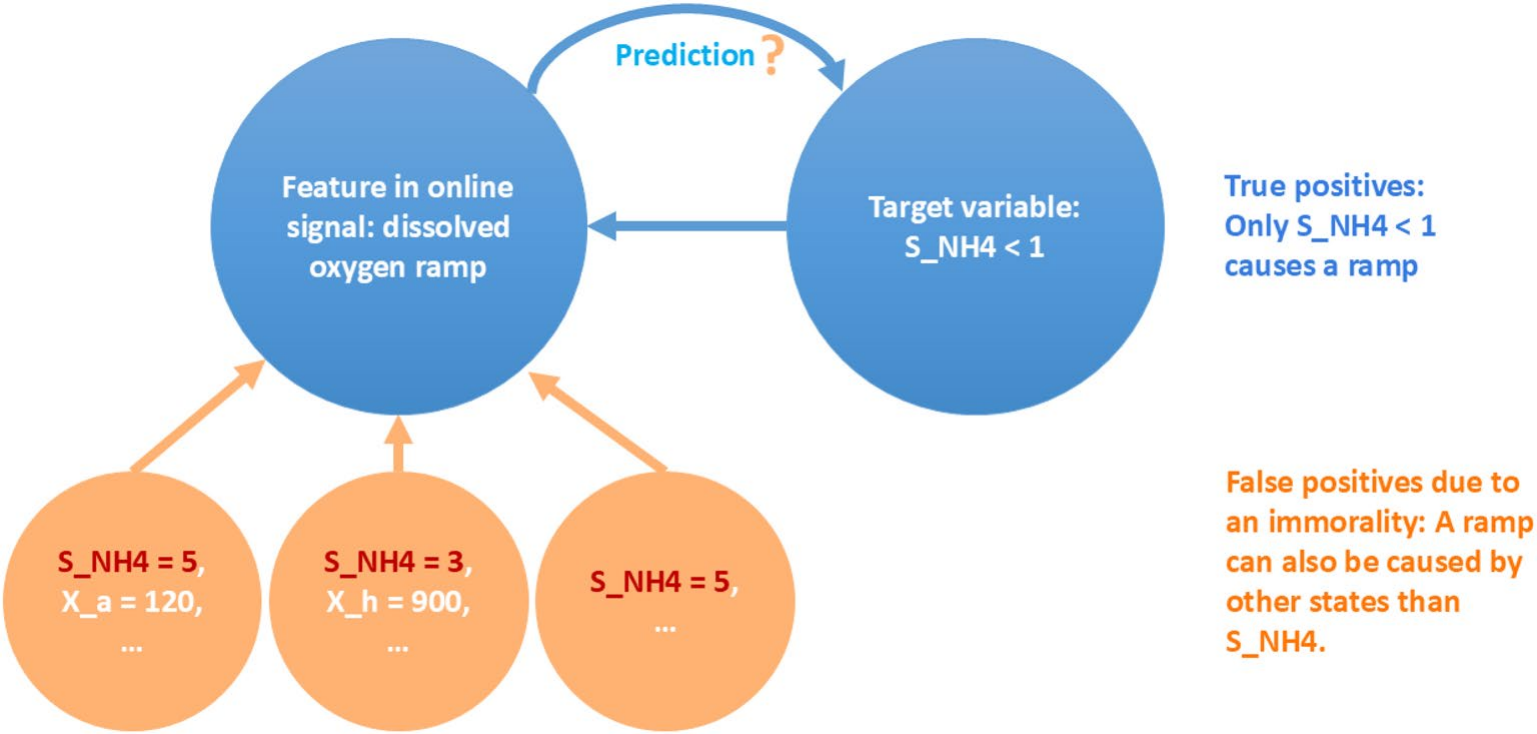
Schematic explanation of immorality, true and false positive predictions. $S_\text {NH4}$ represents the target variable in this case, the ammonium concentration. $X_\text {a}$ (the autotrophic bacteria) and $X_\text {h}$ (the heterotrophic bacteria) are states involved in an immorality and could lead to false predictions. This can occur with any other state variable, excluding the target variable, that is used to predict the feature.

Schneider et al.^16^ directly compared a soft sensor to predict complete ammonia oxidation using a ramp feature (i.e. a non-saddle inflection point, see ”Ramp” in Fig. 7) in the dissolved oxygen signal based on data from maintained and unmaintained sensors. The ammonium-depletion detection based on the ramp feature are robust by design to most effects from noise, measurement uncertainties, or effects of sensor wearing by relying on the qualitative trend instead of absolute values and including a low-pass filter. Nevertheless, three time-consuming sampling campaigns revealed at least two alternative causes for the dissolved-oxygen ramp feature i.e. the aeration pattern and alkalinity limitation. Additionally, in silico experiments based on synthetic data generated with SUMO^18^ revealed that short solids retention time^6^ can cause a false predictions (see Fig. 1. Hence, designing robust soft sensors is constrained by our ability to systematically explore the causes of the feature, e.g. due to the plethora of state combinations that can lead to it. Moreover, the presence of alternative causes for the feature (see the supplementary information sec.5) poses a risk of erroneous soft-sensor predictions, potentially affecting human and environmental health. To avoid these undesired effects, a systematic identification of the alternative causes and the analysis of the plausibility of their occurrence is required.

For complex systems such as the human brain, global weather, or biochemical wastewater treatment processes, understanding the functional architecture is crucial for defining effective, rigorous, principled, and general methods, yet remains a challenge^19^. To find such a method, we systematically analyse sets of Ordinary Differential Equation (ODEs) using an approach that we call Dynamical Systems Analysis (DSA, see^20^, for a joyful introduction). The approach aims at understanding a set of ODEs without integrating them, usually leading to efficient workflows and adding a novel perspective to problem-solving. DSA encompasses techniques for characterising stability and bifurcations (cf. tipping points), and allows screening any dynamic model for a plethora of features. In other fields, such as robotics, DSA is frequently employed (e.g.^21–23^) to gain mathematical insights into the engineering problem at hand, which more often than not leads to novel solutions. In climate science, DSA has been used to predict abrupt changes like tipping points^24^. DSA also provides an alternative perspective into model calibration^25^, ch.1], which focuses on validating structural features of the model with experimental data, as opposed to the more conventional approach of matching time series. Due to all these properties, we hypothesise that an improved understanding of model dynamics with DSA is crucial for engineering features for robust monitoring and control.

In wastewater treatment, DSA remains an uncommon approach. When DSA is employed, it is frequently referred to with ambiguous terms like ”analysis”, which hinders identifying relevant studies. We, therefore, believe that a review on DSA applied in biochemical treatment and recovery processes of wastewater and similar streams is timely, especially to the popular Activated Sludge Model (ASM) ^26^ and Anaerobic Digestion Model (ADM) ^27^. Hence, we provide a brief one in Table 1.

**Table 1:** Literature review on DSA in anaerobic digestion and activated sludge modelling for wastewater treatment.

Reference	Model	DSA outcome	Implication/application
Vanrolleghem^28^	ASM1 and solid flux sedimentation model	Implication of degradation and settling for control design	Improved control design
Grognard and Bernard^29^	Two-population model for anaerobic processes	Stability analysis with non- trivial solutions	Avoiding instability e.g. acidification
Shen et al.^30^	Anaerobic digestion	Identified three stable points, and a saddle-node bifurcation under typical operation conditions	Identification of stable operating conditions
Nelson and Sidhu^31^	ASM1	Identified two branch points	Determination of optimal residence time
Volcke et al.^32^	Two-step biological conversion system	Identification of steady state multiplicity	Experimental design
Benyahia et al.^33^	Two-step ADM	Bifurcation and stability analysis	Experimental design for a soluble microbial products monitoring strategy
Bornhöft et al.^34^	ADM1	Bifurcation analysis	Model selection: Impact of model simplification
Dionisi et al.^35^	Two-population model in a batch process	Stability analysis	Accelerate calculations to reach steady state
Wade and Wolkowicz^36^	Impulsive annamox	Bifurcation analysis	Prediction of wash-out of nitrite-oxidising bacteria
Neto et al.^37^	Benchmark Simulation Model (BSM) 1	Structural controllability and observability	Stability, controllability and observability
Sampaio et al.^38^	ASM1 extended for greenhouse-gas emissions, two-step nitrification, four-step denitrification, and 10-layer settling	Structural analysis	Full-state controllability and observability of greenhouse gas emissions

These successful examples from the water sector indicate the value of DSA for analysing steady states and stability. These analyses contribute to the identification of regions of optimal operation, control, or observability, and experimental design. However, the use in other fields shows, that the potential of DSA is far from exhausted in the water sector. We deduce that systematic detection of information-rich system dynamics (i.e. features) merits further attention, with applications in feature-based model calibration and feature engineering for soft-sensor development, the latter being the primary focus of this study.

## Results and discussion

Herein, we showcase DSA for qualitative trend feature-based soft-sensor development. To model the activated sludge process we used the mass balance-corrected version of ASM1^39^  by Hauduc et al.^40^. We exemplify the use of DSA for feature engineering by systematically investigating factors affecting the ramp feature in the dissolved oxygen signal in a Sequencing Batch Reactor (SBR) to predict the effluent ammonium concentration. The current work complements previous data-driven, mechanistic, or hybrid feature-learning approaches ^41^ on a process familiar to the authors to introduce and discuss DSA as a potential standard procedure for soft-sensor development.

### Model dependency graph

**Fig. 2:**
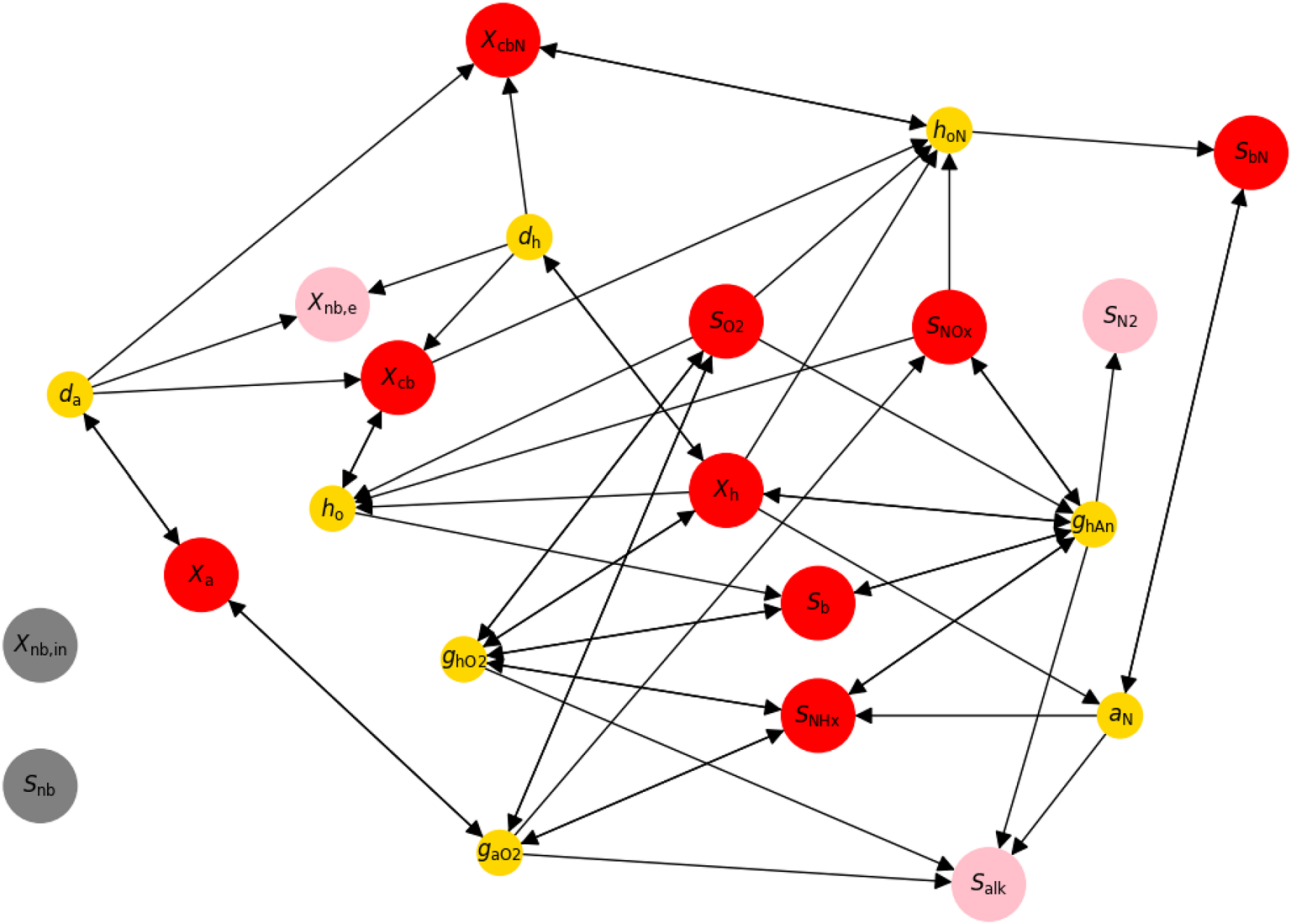
ASM1 state dependency graph. The graph depicts the interactions between the states in the model. States (larger nodes) affect rates (smaller nodes), which in turn affect the states through their time derivative. The arrows indicate which states and rates affect each other. Pink nodes with no child are measurements which do not impact other states. Grey nodes with neither parent nor child are uncoupled (i.e. the non-biodegradable organics, $S_\text {nb}$, and particulate non-biodegradable organics from the influent, $X_\text {nb,in}$). The red nodes are the potential alternative causes for the ramp feature that would lead to a false prediction of the target variable.

A worthwhile first step to get an overview before performing a DSA is a visual inspection of the dependencies. The dependency graph implies causation from the relations between states defined in the analysed model. Figure 2 shows the dependency graph of the 14 states of the mass balance-corrected ASM1 Hauduc et al.^40^. The many circular dependencies reflect the high complexity of the system. Furthermore, all processes follow the pattern state $\rightarrow$ rate $\rightarrow$ state, which hints at the specific model structure presented in Section “ Identification of states leading to dissolved oxygen ramps”. By looking at the arrows and their direction, we can identify that five of these states do not affect the dynamics of the other states, of which two are uncoupled (no children or parents in the graph, i.e. $X_\text {nb,in}$, $S_\text {nb}$), and three are measurements (no children, i.e. $X_\text {nb,e}$, $S_\text {N2}$, $S_\text {alk}$). Therefore, the dynamics of ASM1 are determined by the following nine states (in brackets is the notation by Henze et al.^39^):

$S_\text {b}$: Soluble biodegradable organics (SS)
$X_\text {cb}$: Particulate and colloidal biodegradable organics (XS)
$X_\text {h}$: Ordinary heterotrophic organisms (XBH)
$X_\text {a}$: Autotrophic nitrifying organisms (NH4+ to NO3-, XBA)
$S_\text {O2}$: Dissolved oxygen (SO)
$S_\text {NOx}$: Nitrate and nitrite (NO3 + NO2) (considered to be NO3 only for stoichiometry, SNO)
$S_\text {NHx}$: Ammonia (NH4 + NH3) (SNH)
$S_\text {bN}$: Soluble biodegradable organic N (SND)
$X_\text {cbN}$: Particulate and colloidal biodegradable organic N (XND)

### The dissolved oxygen ramp feature in detail

**Fig. 3:**
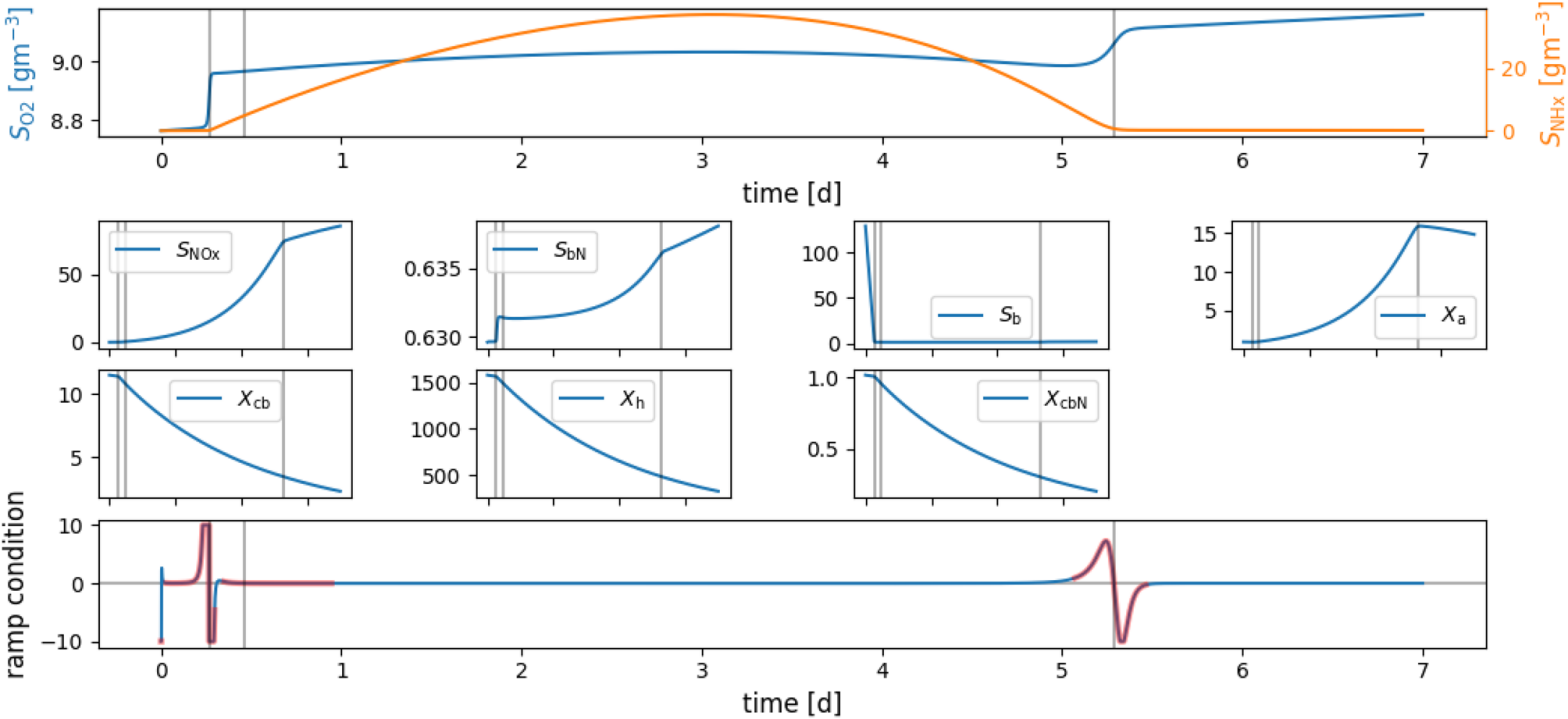
The top panel shows the dissolved oxygen ($S_\text {O2}$) and the effluent ammonium ($S_\text {NHx}$) concentration signals. The ramp time instances are marked on all panels as vertical lines and the time intervals on the x-axes are the same. The second ramp (close to 0.5d) is a false positive for the effluent ammonium concentration estimation, the third one (close to 5.25d) is a true positive feature, and the first one (close to 0.25d) is a true positive that is not caused by the ammonium reaching a concentration below the threshold at the ramp-occurrence time, but the ammonium is already low before the ramp. Possible causes for the ramp feature can be identified by looking at the other panels, showing other state variables of the model (see list in page 5). Each of these three ramp observations corresponds with one point on an inflection point curve. The time series are generated for one single set of initial conditions, and are shown here to display the ramp feature detection. These time series would not be simulated when DSA is used to obtain the inflection point curves. The units of all state variables are $gm^{-3}$.

To illustrate the traditional way of finding inflection points (i.e. ramp features), we show in Fig. 3 the time-series of the states of ASM1 generated by one set of initial conditions. With $S_\text {O2}$ and $S_\text {NHx}$ in the top panel true and false positive (see Fig. 1 for a definition) ramp features can be identified in this single batch run. The vertical lines represent time instances where the conditions of a ramp in the oxygen signal (defined in Eqs. (9)-(10)) are satisfied.

The first ramp in the $S_\text {O2}$ signal is particularly interesting. If the soft sensor does not identify this ramp correctly to stop the treatment process, the ammonium concentration starts rising. The relevant process is the ammonification during the decay of heterotrophic bacteria in the absence of sufficient autotrophic bacteria. The second ramp is a false positive one. The third ramp is what we generally would expect in a biochemical treatment process that recovers wastewater batch-wise. Remarkable is also that the two true positive ramps have a much steeper slope than the false positive one. The first objective of the present work is to use DSA to efficiently screen for potential causes of false positives in the ramp feature, in other words to identify when the soft sensor fails to estimate the ammonium concentration correctly. Another objective is to explore the potential of DSA to make the ramp feature more robust.

Formally, ramps are zeros of the second time derivative in which the first derivative is positive (i.e. non-saddle inflection point). The locus, i.e. the set of all points that satisfy the specified conditions, of ramps can be described mathematically by:

1 $$\begin{aligned} \text {ramps} {:}{=}\left\{ t : \dot{x}(t) > 0\, \wedge \, \ddot{x} = 0 \right\} \end{aligned}$$

where the signal is given by *x*(*t*), the doted symbols represent time derivatives, and the wedge ($\wedge$) is the logical conjunction (and) symbol, meaning that both criteria need to be fulfilled simultaneously.

### Identification of states leading to dissolved oxygen ramps

To identify the states that lead to a ramp feature in the dissolved oxygen signal (represented by the state $S_\text {O2}$), we need to compute the second time derivative of this state (i.e. $\ddot{S}_\text {O2}$), see Eq. (13). This derivative can be computed directly from the model equations without having to simulate the full set of equations to generate a signal, making DSA a compelling and computationally efficient method. This computation can be done for any model structure, but for the ASM family the computations are simplified thanks to the structure of the model that can for example be identified from Fig. 2.

2 $$\begin{aligned} \dot{\boldsymbol{x}}&= {\textsf{M}}\, \boldsymbol{r}(\boldsymbol{x}) + \boldsymbol{z}\end{aligned}$$

3 $$\begin{aligned} \ddot{\boldsymbol{x}}&= {\textsf{M}}\,\dot{\boldsymbol{r}} + \dot{\boldsymbol{z}} = {\textsf{M}}\,{\textsf{J}}_{\boldsymbol{r}}\, \dot{\boldsymbol{x}} + \dot{\boldsymbol{z}} \end{aligned}$$

where we used boldface, e.g. $\boldsymbol{x}$, for vectors, and capital typewriter font, e.g. ${\textsf{M}}$ for matrices. In Eq. (2), $\boldsymbol{x}$ is the vector of states of the model. The states are then transformed via the mapping $\boldsymbol{r}(\boldsymbol{x})$, into a new vector, which herein we will call *rates* vector. The matrix ${\textsf{M}}$, such as the stoichiometric matrix by Gujer and Henze^42^, combines the rates to produce the time derivatives of the states. The additional term ($\boldsymbol{z}$) in Eq. (2) represents the *actuation* on the states of the system. More details and explanations are given in section “Model structure”. Therefore, if the autonomous parts of the model, ${\textsf{M}}$ and $\boldsymbol{r}(\boldsymbol{x})$, are given in tabular form, we can evaluate Eqs. (2) and (3) directly, which allows to analyse any features based on the time derivatives of the model states.

Applying these formulae to ASM1 (given in supplementary information section 4), we obtain equations for the first and second time derivative of the dissolved-oxygen state ($S_\text {O2}$). For the first derivative, we get:

4 $$\begin{aligned}&\begin{aligned} \dot{S}_\text {O2}&= m_{S_\text {O2}\,g_\text {aO2}}\, g_\text {aO2} {\left( S_\text {NHx},S_\text {O2},X_\text {a} \right) } + m_{S_\text {O2}\,g_\text {hO2}}\, g_\text {hO2}{\left( S_\text {NHx},S_\text {O2},S_\text {b},X_\text {h} \right) } + z_\text {O2} \end{aligned}\end{aligned}$$

5 $$\begin{aligned}&m_{S_\text {O2}\,g_\text {aO2}} :=\frac{\gamma _\text {a} + \iota _\text {COD,NO3}}{\gamma _\text {a}}\quad m_{S_\text {O2}\,g_\text {hO2}} :=\frac{\gamma _\text {h} - 1}{\gamma _\text {h}} \end{aligned}$$

6 $$\begin{aligned}&g_\text {aO2}{\left( S_\text {NHx},S_\text {O2},X_\text {a} \right) } :=\mu _\text {max,a}\, X_\text {a} \frac{S_\text {NHx} S_\text {O2}}{\left( S_\text {NHx} + \kappa _\text {NHx,a}\right) \left( S_\text {O2} + \kappa _\text {O2,a}\right) }\end{aligned}$$

7 $$\begin{aligned}&g_\text {hO2}{\left( S_\text {NHx},S_\text {O2},S_\text {b},X_\text {h} \right) } :=\mu _\text {max,h}\, X_\text {h} \frac{S_\text {NHx} S_\text {O2} S_\text {b}}{\left( S_\text {NHx} + \kappa _\text {NHx,h}\right) \left( S_\text {O2} + \kappa _\text {O2,h}\right) \left( S_\text {b} + \kappa _\text {b}\right) } \end{aligned}$$

Since the biological activity of heterotrophic and autotrophic bacteria consumes dissolved oxygen, the first two terms are always non-positive. The signs of $m_{S_\text {O2}\,g_\text {aO2}}$ and $m_{S_\text {O2}\,g_\text {hO2}}$ are determined by the coefficients in Eq.  (5), and for plausible values of $\gamma _a$ and $\gamma _h$ (see in the supplementary information tab.3), these signs are negative.

In the equations above we explicitly show the dependency of the rates (states in parentheses) that we extracted from the model equations. We omit them in the formulae for the second derivative:

8 $$\begin{aligned} \begin{aligned} \ddot{S}_\text {O2} =\,\,&\dot{S}_{\text {NHx}}\, \partial _{S_\text {NHx}}\left( g_\text {aO2}\, m_{S_\text {O2}\,g_\text {aO2}} + g_\text {hO2}\, m_{S_\text {O2}\,g_\text {hO2}}\right) \\ +\,\,&\dot{S}_{\text {O2}}\, \partial _{S_\text {O2}} \left( g_\text {aO2}\, m_{S_\text {O2}\,g_\text {aO2}} + g_\text {hO2}\, m_{S_\text {O2}\,g_\text {hO2}}\right) \\ +\,\,&\dot{S}_{\text {b}} \partial _{S_\text {b}}g_\text {hO2}\, m_{S_\text {O2}\,g_\text {hO2}} + \dot{X}_{\text {a}} \partial _{X_\text {a}}g_\text {aO2}\, m_{S_\text {O2}\,g_\text {aO2}} + \dot{X}_{\text {h}} \partial _{X_\text {h}}g_\text {hO2}\, m_{S_\text {O2}\,g_\text {hO2}} \\ +\,\,&z_\text {O2}\, \partial _{S_\text {O2}}\left( g_\text {aO2}\, m_{S_\text {O2}\,g_\text {aO2}} + g_\text {hO2}\, m_{S_\text {O2}\,g_\text {hO2}}\right) + \dot{z}_\text {O2} \end{aligned} \end{aligned}$$

The conditions for the ramp (section “Model signal features”) read:

9 $$\begin{aligned}&\dot{S}_\text {O2} > 0\end{aligned}$$

10 $$\begin{aligned}&\ddot{S}_\text {O2} = 0 \end{aligned}$$

The first condition for a ramp, Eq. (9), requires a positive time derivative. This expresses that the rate at which oxygen is transferred to the liquid phase ($z_\text {O2}$) needs to be bigger than the oxygen uptake rate by the bacteria, as there cannot be more oxygen consumed than is present. Therefore, in ASM1 the dissolved oxygen ramp is a feature that can only be generated with a source of dissolved oxygen (aeration) as otherwise Eq. (9) cannot be fulfilled. This underlines the role of aeration for the detection and robustness of the ramp feature, which in a different context, was observed in a real-world case in^6^.

Using DSA shows that the conditions (9) and (10) depend on other states besides $S_\text {NHx}$ and $S_\text {O2}$, i.e. $S_\text {NOx}, S_\text {bN}, S_\text {b}$, $X_\text {a}, X_\text {cb}, X_\text {h}$. So, even if we kept $S_\text {NHx}$ constant at a value above the prediction threshold for a full ammonium depletion, changes to these other states could generate a ramp that is not due to a $S_\text {NHx}$ below this threshold. This can potentially lead to false positive predictions with a ramp-based soft sensor.

### Immorality of the dissolved oxygen ramp

**Fig. 4:**
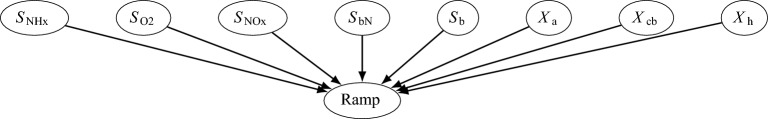
Causes of the dissolved oxygen ($S_\text {O2}$) ramp feature. Obtained from the variables in Eq. (8). Several V-structures are identified from the causal relations. See list in page (section “Model signal features”) for the variable names.

From the nine states in the dependency graph (Fig. 2), DSA shows that eight states are actual causes of a ramp, $X_\text {cbN}$ is not a cause. These eight states form several V-structures within the causal diagram of the ramp feature in Fig. 4; known as *immoralities*^43^. The states (parents) in a V-structure, will be associated (correlated) when data is selected conditional on the presence of the feature (child). These correlations can be exploited by a data-driven machine learning model to infer difficult-to-monitor states, e.g. $S_\text {NHx}$ from $S_\text {O2}$, or to alleviate an effluent ammonium prediction task. However, since pure data-driven approaches are limited to exploiting correlations without guaranteeing causality; adding causal relations, as we do here, can enhance the performance and information extracted from the method, e.g. to include root cause analysis and therewith improve the robustness of the sensor.

**Fig. 5:**
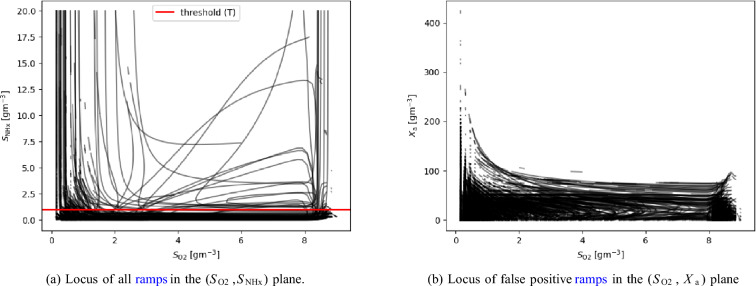
Inflection point curves in two planes. The lines show the combinations of $S_\text {O2}$ and $S_\text {NHx}$ or $S_\text {O2}$ and $X_\text {a}$ that lead to a ramp feature. 10,000 simulations with different initial conditions for each panel were conducted. The darker the area, the more overlapping inflection points are in the region. The ammonium concentrations for (**b**) were restricted to only show panels with false positive ramps i.e. the ammonium concentration is above an arbitrary threshold of 1 ${\rm gm}^{-3}$.

DSA allows the systematic evaluation of conditions that lead to a ramp in a search process by sampling the states independently to determine the causes (parents) of the ramp feature, see Fig. 4. This is illustrated in Fig. 5a which shows all ramps found on the $(S_\text {O2}$, $S_\text {NHx})$ plane for 10,000 uniformly distributed random values of all the other six states that can cause ramps (see supplementary information section 1 for explanations on how to read these curves). Each line is a 0-level set curve of equation (10) that satisfies Eq.  (9). Figure 5 a further shows that many of these ramps are below an arbitrary threshold of 1${\rm gm}^{-3}$ $S_\text {NHx-N}$. All the ramps below the threshold would lead to the correct conclusion that the ammonium oxidation process is complete (true positive), hence the feature is informative. However, many other ramps are clearly above the threshold, and would lead to an erroneous conclusion (false positive).

To further investigate these false positives, Fig. 5b shows all ramps found on the ($S_\text {O2}$, $X_\text {a}$) plane for the autotrophic bacteria. We chose the autotrophic bacteria because they are responsible for the removal of nitrogen compounds such as ammonium. Therefore, we have only sampled values of $S_\text {NHx}$ above the arbitrary threshold, between 1gm-3 to 200${\rm gm}^{-3}$. For all other states, we used the same ranges as before. Hence, only false positive ramps are shown in this plot. This allows us to evaluate the causes of false positive results and identify measures to increase accuracy. The pattern clearly shows that a high concentration of $X_\text {a}$ could drastically reduce the false positive results.

In summary, Fig. 5a and 5b illustrate how DSA can be used to systematically scan a feature space for the occurrence of features and their causes. Analysing the equations allows us to identify causal relations, which is very different from checking data for correlation. For an extended discussion of these results concerning experimental work in^6,16^ see supplementary information sec. 6.

### Ramp points distribution

**Fig. 6:**
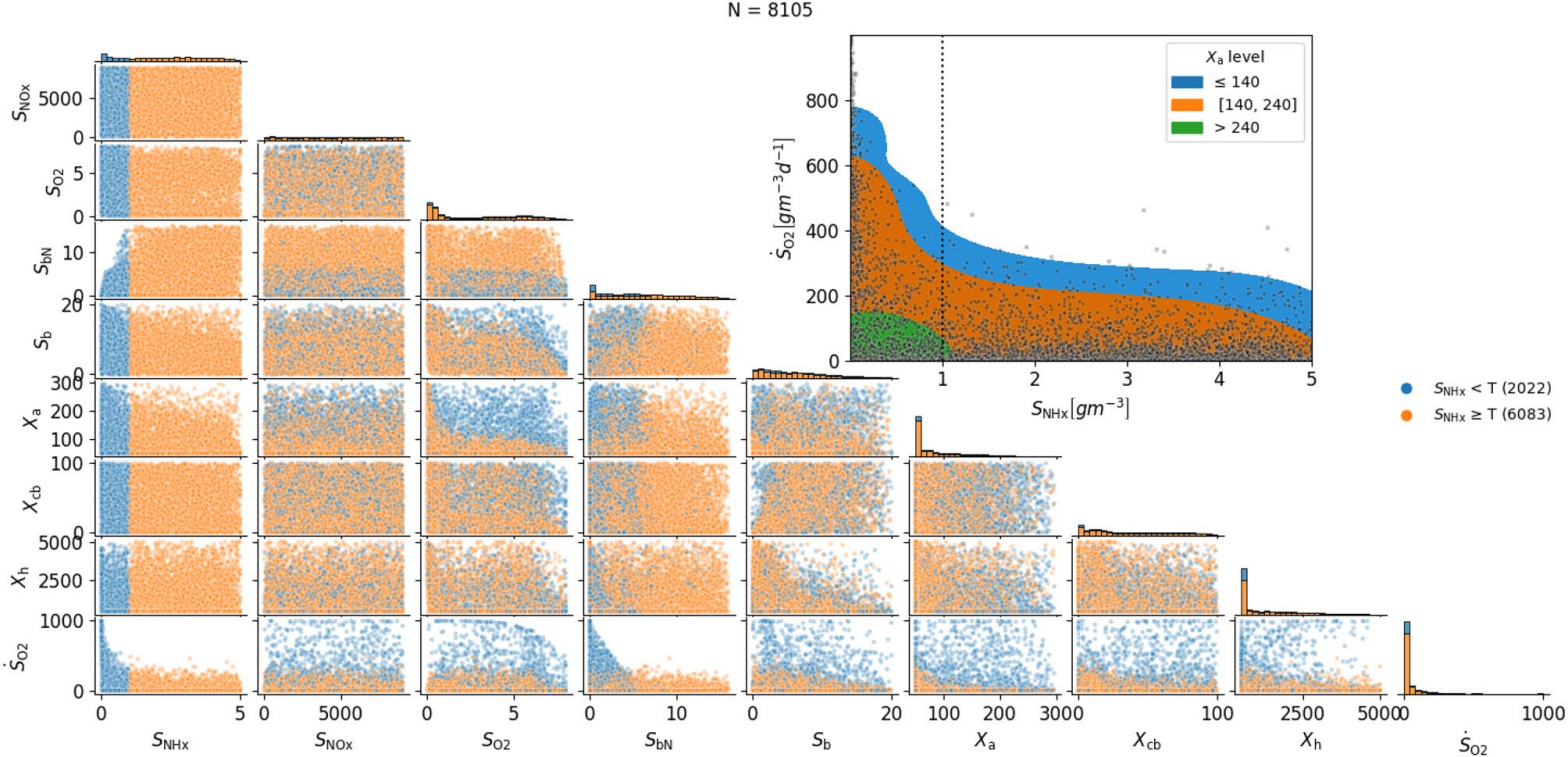
ASM1 ramp points distribution. Distribution of states fulfilling the ramp conditions (9) and (10). The points are colored by whether the value of $S_\text {NHx}$ is above (orange) or below (blue) an arbitrary threshold (T). The number of samples (N) in each category is shown in the legend. The panels on the diagonal always show the density of points. The bottom row of the panels shows the value of the dissolved oxygen time derivative (ramp’s slope). The top-right panel shows the relation between the ramp’s slope and the ammonium concentration. The coloured regions on that panel show the level of autotrophic bacteria ($X_\text {a}$), illustrating a further correlation that can be exploited for increasing the accuracy of a full-ammonium-depletion detection soft sensor.

Figure 6 shows the distribution of states that cause a ramp feature (satisfy Eqs. (9, 10)). Blue and orange dots indicate effluent ammonium concentrations below and above an arbitrary threshold (T), respectively. Blue dots are thus true positives for the ammonium soft sensor and orange dots represent false positives (compare Fig. 1). The plot also shows the dissolved oxygen time derivative ($\dot{S}_\text {O2}$) at these points. We observed in Fig. 3 that true ramps are associated with higher derivatives than false ramps. The top right panel of Fig. 6 ($S_\text {NHx}$ vs $\dot{S}_\text {O2}$) supports this observation, as almost no false ramps are found above dissolved oxygen time derivatives of 400${\rm gm}^{-3}d^{-1}$ Therefore, using time derivative information (slope in a time series plot) together with the detection of ramps constitutes a more accurate feature.  

This observation also provides a plausible explanation for the improved accuracy of an ammonium depletion classifier when slope information was included. Without a restriction on the slope the feature was not informative^6,16^. In these previous studies this slope restriction was found with trial and error, while DSA offers a systematic approach. ASM1 already captures this relation, despite missing processes and its relative simplicity compared to other activated sludge models. Identifying such a slope dependence shows that for the ramp-feature design ASM1 is structurally useful. Therefore, we demonstrate that and how DSA can be used for structural model verification.

In the top right panel of Fig. 6 we further observe that eliminating the false positives by defining a minimal slope and rejecting all ramps below the chosen slope (e.g. $< {400}{\rm gm}^{-3} d^{-1}$) may result in many false negatives. The coloured shaded regions for different levels of $X_\text {a}$ indicate that with a higher $X_\text {a}$ the slope tolerance could be considerably lowered to reduce the false negatives. Hence, finding another feature or sensor to estimate $X_\text {a}$ would increase the accuracy of the ramp-based soft sensor.

Further patterns can be recognised in Fig. 6—For example if $S_\text {bN}$ is above approximately ${7}{\rm gm}^{-3}$ , no correct predictions may be possible. It is reasonable to think that if the soluble biodegradable organic nitrogen is high, too much ammonium is formed, and its concentration drop is limited. The experimental confirmation of this discovery is part of future work. Furthermore, observing multiple additional patterns in Fig. 6 to the ones discussed in this article shows great potential for future studies, e.g., to further improve the soft sensor with an estimate $X_\text {a}$ or to identify an early warning feature for loss of nitrifying biomass. Additionally, the results of the DSA hint at which regions could be used by data-driven models.

Herein, we demonstrated how DSA can be used to improve the feature, e.g. by adding further constraints such as the slope of $S_\text {O2}$. DSA could also be used to discover feature sets from signals of other sensors than dissolved oxygen. These feature sets could further improve the accuracy and robustness of an ammonium-depletion soft sensor or soft sensors for different quantities of interest.

We provide all the code under a GNU free public license in Carbajal and Schneider^44^, hence readers can adapt it to their own analyses and for different models, as long as these models have the structure described in Eq. (3).

### Limitations and outlook

As illustrated herein, DSA is applied to a specific model, and is restricted by the model’s structure. For example, there is another known state variable that could cause a ramp: the alkalinity^45^, which does not influence other states in the definition of ASM1. Hence, no DSA performed on ASM1 will inform us of the influence of alkalinity. However, the presented method for DSA can be applied to other models such as ASM3 to assess the relationship between alkalinity and the ramp feature. In the present work, it was decided to develop a proof of concept of the method based on ASM1 as this is still the most commonly used model in the ASM family.

DSA deals with deterministic dynamics, therefore process noise cannot be handled easily with the current approach. Measurement noise does not affect the dynamics, and therefore correlations between state variables and features based on them are not affected by measurement noise. Detection of these features in real systems, by means of sensors and state estimation, is however affected by measurement noise. Examining the impact of measurement noise and the feasibility of applying DSA results in practical applications is not the focus of this work, nor is it an inherent part of DSA analysis; these are additional analyses conducted subsequently.

## Conclusion

1. We used DSA to systematically evaluate all the mathematically possible solutions leading to a ramp feature in the dissolved oxygen signal in ASM1. The dissolved oxygen ramp is clearly immoral, meaning that not only a low value of the state $S_\text {NHx}$, i.e. an ammonium depletion, can cause the ramp feature, but also eight alternative causes, i.e. $S_\text {O2}$, $S_\text {NOx}$, $S_\text {bN}$, $S_\text {b}$, $X_\text {a}$, $X_\text {cb}$, $X_\text {h}$, $X_\text {cbN}$.
2. With DSA we identified that the slope of the dissolved oxygen at the locus of the ramp is relevant and can increase the accuracy of the ramp feature.
3. Many models (e.g. ASM family or ADM1) follow the same matrix structure which means that the formulae presented here can be applied ”as-is” for a plethora of models in the water and resource recovery domain.
4. The analysis underlines how important systematic falsification studies are, especially when methods are developed for setups that can directly impact human and environmental health such as water recovery facilities for reuse purposes. With DSA, we suggest a concrete method for such a systematic analysis by increasing the robustness of soft sensors through the exposure of immoralities that can then be mitigated by multi-feature approaches.

## Materials and methods

### Dissolved oxygen feature

**Fig. 7:**
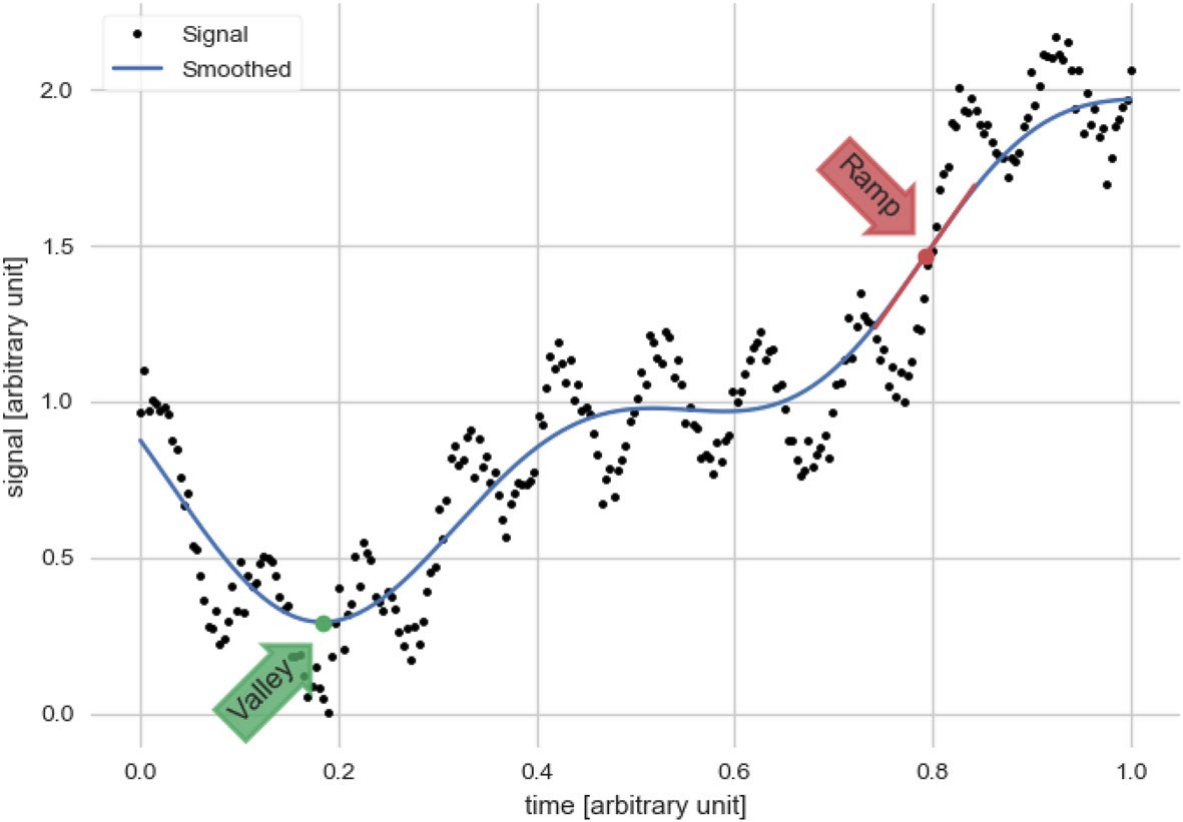
Illustration of two different features based on time derivatives of a signal. An extrema (valley) and a non-saddle inflection point (ramp). The latter we use in this article. An example for the arbitrary unit on the x-axis would be day on the y-axis it could be ${\rm gm}^{-3}$. The figure is created with Carbajal and Schneider^47^.

As introduced in section “Introduction”, a feature can be used to monitor a biochemical wastewater treatment process. From the two measurement signals pH and dissolved oxygen from a previous study^16^, we here chose the latter, as $S_\text {O2}$ is a state in the ASM1 while pH is not a state variable in any of the ASM models. This allowed us to start with a model with a small number (eight) of equations to implement. The feature of the dissolved oxygen is a ramp (see Fig. 7) that can be used to predict the effluent ammonium ($S_\text {NHx}$) concentration. Olsson and Andrews^46^ describe the ramp feature for a plug flow reactor, hence w.r.t. the location. Following the same principle in our study, we use an SBR, thus the ramp is w.r.t. time, i.e. whenever an inflection point is observed during the aeration phase, we assume that the ammonium is fully oxidised so the ammonium nitrogen is below or equal to an arbitrary threshold of ${1}\,\,{\rm gm}^{-3}$ ^6^. If there is no ramp feature the effluent ammonium concentration is estimated to be above ${1}\,\,{\rm gm}^{-3}$. For this prediction to be accurate and robust there should be no other state than an ammonium concentration below or equal to ${1}\,\,{\rm gm}^{-3}$ leading to the ramp feature. First, we obtain all the mathematically possible solutions for a ramp feature in ASM1 with DSA. See section “Model signal features” for a detailed description of how we obtained these solutions. Next, we further constrain these mathematically possible solutions to the physically plausible ones by restricting them to only positive states. Restrictions were that concentrations cannot be negative and the setting of a wide, yet feasible range for all state variables. See tabs. 4-6 in the supplementary information for ranges reported in the literature, which we consulted to restrict the phase space of the state variables.

### Model signal features

We are concerned with computing signal features based on the signal’s derivatives w.r.t. time (time derivatives). Figure 7 shows two examples of such features: a valley, which is a minimum extreme point and a ramp. Extrema are zeros of the signal’s first time derivative, and ramps are zeros of the second time derivative in which the first derivative is positive (i.e. non-saddle inflection point). The locus (i.e. set of all points that satisfies the specified conditions) of ramps can be described mathematically by Eq. (1).

Equation (1) can be used with signals that are differentiable twice. Also, we exclude saddle-points by requiring that the first time derivative is positive. The definition of ramp includes the concavity changing points of sigmoid functions like the hyperbolic tangent (as shown in Fig. 7) and points at which the signal looks locally like a straight line (i.e. proportional to time), even if its concavity does not change sign.

Therefore, by obtaining an expression of the first and second time derivatives of the signal directly from the model, we can compute the locus of all ramps without solving the full differential equations or modelling time series.

### Model structure

We apply our approach to models with the following structure:

11 $$\begin{aligned} \dot{\boldsymbol{x}}&:={\textsf{M}}\, \boldsymbol{r}(\boldsymbol{x}) + \boldsymbol{z}\end{aligned}$$

12 $$\begin{aligned} \boldsymbol{y}&:=\boldsymbol{h}(\boldsymbol{x}) \end{aligned}$$

where we used boldface, e.g. $\boldsymbol{x}$, for vectors, and capital typewriter font, e.g. ${\textsf{M}}$ for matrices. In Eq. (11), $\boldsymbol{x}$ is the vector of states of the model, which herein we take to be a real column matrix, i.e. $\boldsymbol{x} \in \mathbb {R}^{N\times 1}$ ($N$ is the number of states, e.g. $N=13$ in the original ASM1, Henze et al.^39^). The states are then transformed via the mapping $\boldsymbol{r}(\boldsymbol{x})$, into a new vector, which herein we will call *rates* vector and, by slight abuse of notation, denote also $\boldsymbol{r}$. This transformation might not preserve the dimension, so the resulting rates vector might have a different dimension $\boldsymbol{r}(\boldsymbol{x}) \in \mathbb {R}^{R\times 1}$ ($R$ is the number of reaction rates, e.g. $R=8$ in the original ASM1,  Henze et al.^39^).

The matrix ${\textsf{M}}$ combines the $R$ rates to produce the $N$ time derivatives of the states, $\dot{\boldsymbol{x}}$. Hence we have ${\textsf{M}} \in \mathbb {R}^{N\times R}$. The additional term ($\boldsymbol{z}$) in Eq. (11) represents the *actuation* on the states of the system (Please note: the actuation might not be the inputs defined by a controller, a further *input model* ($\boldsymbol{b}$) might be needed: $\boldsymbol{z} :=\boldsymbol{b}(\boldsymbol{u})$, where $\boldsymbol{u}$ are the controllable inputs and $\boldsymbol{z}$ are the actuations).

Equation (12) (a.k.a. *measurement model* ^48^,ch.4.3]) represents a mapping that generates measurement signals, $\boldsymbol{y}$ (modelling those that could be produced by a sensor), based on the model states ($\boldsymbol{x}$).

Until here we have omitted the dependence of Eqs. (11, 12) on parameters to keep the notation simple. All the model’s components described till now can depend on parameters (free parameters that must be estimated, e.g. experimentally and fundamental ones).

Many models for activated sludge and anaerobic digestion obey this structure, for example, all the ASM family^26^, and several anaerobic digestions models^49^ such as ADM1^27^. As an example, all model components described above correspond to ASM1 and can be found in the supplementary information section 4, including tab. 3 with all parameters and their description. This means that the mathematical analysis presented here can be applied to any model that follows the form in Eqs. (11, 12) by inserting the matrix and the rates of a different model than ASM1 into the formula.

As the model evolves in time, Eq. (11) provides the first time derivative of the signals. The second time derivative of all states is obtained by direct application of the chain rule:

13 $$\begin{aligned} \ddot{\boldsymbol{x}} = {\textsf{M}}\,\dot{\boldsymbol{r}} + \dot{\boldsymbol{z}} = {\textsf{M}}\,{\textsf{J}}_{\boldsymbol{r}}\, \dot{\boldsymbol{x}} + \dot{\boldsymbol{z}} \end{aligned}$$

where ${\textsf{J}}_{\boldsymbol{r}}$ is the Jacobian of the rates w.r.t. the states, i.e.

14 $$\begin{aligned} {\textsf{J}}_{\boldsymbol{r}} {:}{=}\begin{bmatrix} \nabla _{\boldsymbol{x}}^\top r_1(\boldsymbol{x}) \\ \vdots \\ \nabla _{\boldsymbol{x}}^\top r_R(\boldsymbol{x}) \end{bmatrix} = \begin{bmatrix} \partial _{x_1}{r_1} & \ldots & \partial _{x_N}{r_1} \\ \vdots & \ddots & \vdots \\ \partial _{x_1}{r_R} & \ldots & \partial _{x_N}{r_R} \end{bmatrix} \end{aligned}$$

The ramps of the *i*-th signal in these models is then given by

15 $$\begin{aligned} \text {ramps} {:}{=}\left\{ t : {\textsf{M}}_{i,:}\,\boldsymbol{r} + z_i > 0\, \wedge \, {\textsf{M}}_{i,:}\,{\textsf{J}}_{\boldsymbol{r}}\, \dot{\boldsymbol{x}} + \dot{z}_i = 0 \right\} \end{aligned}$$

### Ramp contingency

Here the typical and the physical minimum and maximum values are presented for all eight relevant states that can cause the $S_\text {O2}$ ramp feature.

For all the evaluations in this article, we used Python 3^50^ and SymPy^51^ (any other computer algebra system could be used), complemented by a minimal amount of manual analysis that will be further automated in the future^44^.

## Supplementary Information

Supplementary Information.

## Data Availability

All data supporting the findings of this study were generated using code available in the public GitLab repository at https://gitlab.com/sbrml/dsa-signal-features/. The repository contains all scripts and instructions necessary to reproduce the datasets and figures presented in this article in the folder doc/examples. The code is available under the GNU Public License Version 3, and can be accessed without restriction.

## Abbreviations

ADM: Anaerobic digestion model
ASM: Activated sludge model
BSM: Benchmark simulation model
DSA: Dynamical systems analysis
ODE: Ordinary differential equation
ramp: A time-based signal feature. It is defined as the signal’s non-saddle (non-zero first time derivative) inflection point(zero second time derivative)
SBR: Sequencing batch reactor
