# Supplementary material for: Unveiling the immoral ramp feature of dissolved oxygen signals with dynamical systems analysis: perspectives for robust soft-sensor development
Source: Sci Rep. 2026 Apr 1;16:15414. doi: 10.1038/s41598-026-43885-y (PMC13184300; doi:10.1038/s41598-026-43885-y)
Supplement: Supplementary file 1 — Supplementary Information. [file 41598_2026_43885_MOESM1_ESM.pdf]

Supplementary information for "Unveiling the immoral ramp feature of dissolved oxygen signals with dynamical systems analysis: perspectives for robust soft-sensor development"

Mariane Y. Schneider*[1,2,3], Elena Torfs[4], and Juan Pablo Carbajal[5]

[1]BIOMATH, Department of Data Analysis and Mathematical Modelling, Ghent University, Coupure Links 653, 9000 Ghent, Belgium
[2]CAPTURE, Centre for Advanced Process Technology for Urban REsource Recovery, Ghent University, Frieda Saeysstraat 1, Ghent, Belgium
[3]DTU Sustain, DTU, Bygningstorvet, building 115, 2800 Lyngby, Denmark
[4]modelEAU, Département de Génie Civil et de Génie des Eaux, Université Laval, Pavillon Adrien-Pouliot, 1065, Av. de la Médecine, QC G1V 0A6, Canada
[5]Institute for Energy Technology, OST Eastern Switzerland University of Applied Sciences, Oberseestrasse 10, Rapperswil, Switzerland

February 2026

1 Time derivatives

Here we detail the calculation for the time derivative of a model with the following structure

$$\dot{\boldsymbol{x}} = \mathtt{M}\boldsymbol{r}(\boldsymbol{x}) \tag{1}$$

to which we introduce a new term in the derivatives

$$\dot{\boldsymbol{x}} = \mathtt{M}\boldsymbol{r}(\boldsymbol{x}) + \boldsymbol{z}(\boldsymbol{x}, t) \tag{2}$$

By direct derivation, the second time derivative reads (we do not show the explicit dependence anymore):

$$\ddot{\boldsymbol{x}} = \mathtt{M}\dot{\boldsymbol{r}} + \dot{\boldsymbol{z}} \tag{3}$$
$$\dot{\boldsymbol{r}} = \partial_{\boldsymbol{x}}\boldsymbol{r}\;\dot{\boldsymbol{x}} \tag{4}$$
$$\dot{\boldsymbol{z}} = \partial_{\boldsymbol{x}}\boldsymbol{z}\;\dot{\boldsymbol{x}} + \partial_t \boldsymbol{z} \tag{5}$$

replacing

$$\ddot{\boldsymbol{x}} = \mathtt{M}\partial_{\boldsymbol{x}}\boldsymbol{r}\;\dot{\boldsymbol{x}} + \partial_{\boldsymbol{x}}\boldsymbol{z}\;\dot{\boldsymbol{x}} + \partial_t \boldsymbol{z} \tag{6}$$

Another approach is to think that the introduction of the derivatives modifies the structure of the system in (1):

$$\dot{\boldsymbol{x}} = \mathtt{M}^*\boldsymbol{r}^*(\boldsymbol{x}) \tag{7}$$

with the new elements being concatenations of the old

$$\mathtt{M}^* = \begin{bmatrix}\mathtt{M} & \mathbb{I}_{\dim \boldsymbol{z}}\end{bmatrix} \tag{8}$$
$$\boldsymbol{r}^*(\boldsymbol{x}) = \begin{bmatrix}\boldsymbol{r}(\boldsymbol{x}) \\ \boldsymbol{z}(\boldsymbol{x}, t)\end{bmatrix} \tag{9}$$

where $\mathbb{I}_{\dim \boldsymbol{z}}$ is the identity matrix of the size of $\boldsymbol{z}$. Expanding:

*Corresponding author: mariane.schneider@ugent.be

$$\mathtt{M}^* \boldsymbol{r}^* = \begin{bmatrix}\mathtt{M} & \mathbb{I}_{\dim \boldsymbol{z}}\end{bmatrix} \begin{bmatrix}\boldsymbol{r} \\ \boldsymbol{z}\end{bmatrix} = \mathtt{M}\boldsymbol{r} + \boldsymbol{z} \tag{10}$$

which shows the equivalence to eq. (2). In this case the second derivative is,

$$\ddot{\boldsymbol{x}} = \mathtt{M}^* \dot{\boldsymbol{r}}^* = \mathtt{M}^* \left(\partial_{\boldsymbol{x}} \boldsymbol{r}^* \ \dot{\boldsymbol{x}} + \partial_t \boldsymbol{r}^*\right) \tag{11}$$

which gives a simple general formula applicable also in the absence of actuation.

To verify equality with the previous derivation we expand the terms. Computing the Jacobian

$$\partial_{\boldsymbol{x}} \boldsymbol{r}^* = \partial_{\boldsymbol{x}} \begin{bmatrix}\boldsymbol{r} \\ \boldsymbol{z}\end{bmatrix} = \begin{bmatrix}\partial_{\boldsymbol{x}} \boldsymbol{r} \\ \partial_{\boldsymbol{x}} \boldsymbol{z}\end{bmatrix} \tag{12}$$

then

$$\partial_{\boldsymbol{x}} \boldsymbol{r}^* \ \dot{\boldsymbol{x}} = \begin{bmatrix}\partial_{\boldsymbol{x}} \boldsymbol{r} \\ \partial_{\boldsymbol{x}} \boldsymbol{z}\end{bmatrix} \dot{\boldsymbol{x}} = \begin{bmatrix}\partial_{\boldsymbol{x}} \boldsymbol{r} \ \dot{\boldsymbol{x}} \\ \partial_{\boldsymbol{x}} \boldsymbol{z} \ \dot{\boldsymbol{x}}\end{bmatrix} \tag{13}$$

and

$$\mathtt{M}^* \partial_{\boldsymbol{x}} \boldsymbol{r}^* \ \dot{\boldsymbol{x}} = \begin{bmatrix}\mathtt{M} & \mathbb{I}_{\dim \boldsymbol{z}}\end{bmatrix} \begin{bmatrix}\partial_{\boldsymbol{x}} \boldsymbol{r} \ \dot{\boldsymbol{x}} \\ \partial_{\boldsymbol{x}} \boldsymbol{z} \ \dot{\boldsymbol{x}}\end{bmatrix} = M \partial_{\boldsymbol{x}} \boldsymbol{r} \ \dot{\boldsymbol{x}} + \partial_{\boldsymbol{x}} \boldsymbol{z} \ \dot{\boldsymbol{x}} \tag{14}$$

The temporal term is

$$\mathtt{M}^* \partial_t \boldsymbol{r}^* = \begin{bmatrix}\mathtt{M} & \mathbb{I}_{\dim \boldsymbol{z}}\end{bmatrix} \begin{bmatrix}\partial_t \boldsymbol{r} \\ \partial_t \boldsymbol{z}\end{bmatrix} = \mathtt{M} \overbrace{\partial_t \boldsymbol{r}}^{\boldsymbol{0}} + \partial_t \boldsymbol{z} \tag{15}$$

Putting all together

$$\ddot{\boldsymbol{x}} = M \partial_{\boldsymbol{x}} \boldsymbol{r} \ \dot{\boldsymbol{x}} + \partial_{\boldsymbol{x}} \boldsymbol{z} \ \dot{\boldsymbol{x}} + \partial_t \boldsymbol{z} \tag{16}$$

which is the same as eq. (6).

1.1 Single state actuation

If we assume that the actuation does not depend directly on time, and only depends in a single state, the actuation vector has the form:

$$\boldsymbol{z}(\boldsymbol{x}, t) = \mathbf{1}_{x_i} z(x_i) \tag{17}$$

where $\mathbf{1}_{x_i}$ is a column vector filled with zeros, except in the location of element x_i. That is, $\boldsymbol{z}$ affects only the state x_i and it depends only on that state.

We have that

$$\partial_t \boldsymbol{z} = \mathbf{0} \tag{18}$$

$$\partial_{\boldsymbol{x}} \boldsymbol{z} \ \dot{\boldsymbol{x}} = \partial_{\boldsymbol{x}} \mathbf{1}_{x_i} z(x_i) \ \dot{\boldsymbol{x}} = \partial_{x_i} z \ \mathtt{1}_{x_i x_i} \dot{\boldsymbol{x}} = \partial_{x_i} z \ \dot{x}_i \ \mathbf{1}_{x_i} \tag{19}$$

where $\mathtt{1}_{x_i x_i}$ is a matrix with a 1 in the (x_i, x_i) element, and zero otherwise. By replacing into eq. (16) we obtain,

$$\ddot{\boldsymbol{x}} = M \partial_{\boldsymbol{x}} \boldsymbol{r} \ \dot{\boldsymbol{x}} + \partial_{x_i} z \ \dot{x}_i \ \mathbf{1}_{x_i} \tag{20}$$

and the x_i component is

$$\ddot{x}_i = M_{x_i:} \partial_{\boldsymbol{x}} \boldsymbol{r} \ \dot{\boldsymbol{x}} + \partial_{x_i} z \ \dot{x}_i \tag{21}$$

These are the formulas used in the software [1] accompanying this publication.

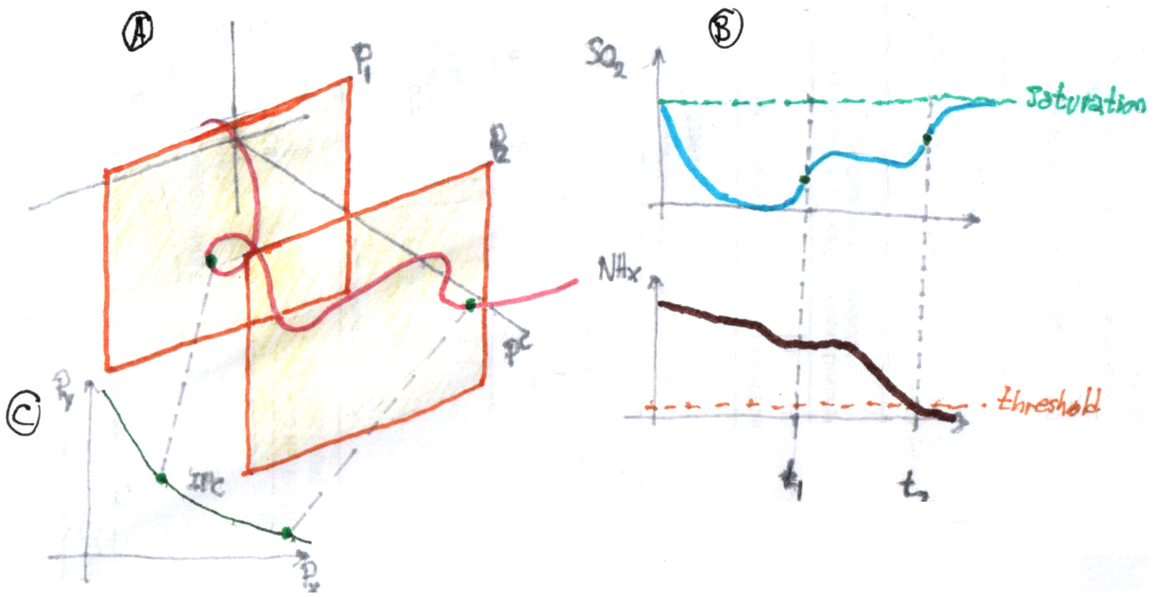

Figure 1: Construction and interpretation of a ramp points curve. A: a trajectory of a dynamical system in $\mathbb{R}^N$. In the drawing, the space is decomposed in two sub-spaces of dimension 2 (the planes are labelled with P in the diagram), and $N-2$ (the perpendicular direction to the planes, the orthogonal complement $P^\perp$ of P). By fixing the values of all components of a vector in $P^\perp$ a plane is defined as shown. The trajectory crosses these planes as it evolves. B: Components of the trajectory can be plotted as a function of time, as an example the states S_{O2} and NH_x from Activated Sludge Model1 (ASM) are shown. The points in which a ramp is realised are marked here and in the trajectory. The time of the ramps have their corresponding planes in panel A, t_1 with P_1, etc. C: By merging all the planes and the points corresponding to a ramp in them, we obtain the locus of all ramps. These loci might look like curves in the obtained plot. The curve collects points from different planes (different values of the states in $P^\perp$)

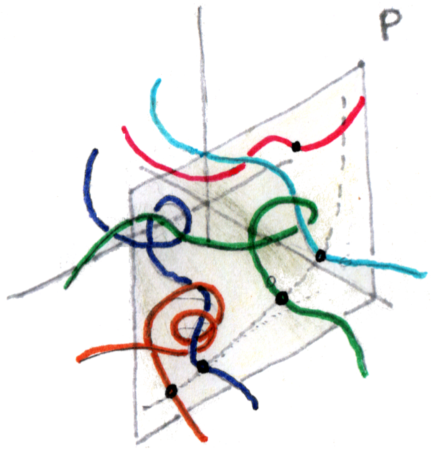

Figure 2: Multiple trajectories crossing the plane P, for fixed values of $P^\perp$. Some trajectories intersect the plane with a ramp in the time signal of the selected component, e.g. S_{O2}, while others do not (pink trajectory). Only those with a ramp contribute to the ramp locus (dashed lines on P).

2 Ramp points curve

The locus of all ramps defines a hyper-surface of dimension $\mathbb{R}^{N-1}$. This surface cannot be visualized easily. Therefore we choose a sub-space of dimension 2 that we call P (for plane) and we collect points fulfilling the ramp conditions in it.

In P we have two states of our choice, e.g. S_{O2} and X_a, and all other states are in the orthogonal complement of P, called $P^\perp$. In fig. 1 we depict $P^\perp$ as a single direction, but the reader should understand that in fact, this sub-space is of dimension $N-2$.

To put a ramp point in P, we must first define a value for all states in $P^\perp$. These are the planes shown in panel A of fig. 1. For any value we choose for these other states, and for any value of the states in P, we can always find a trajectory of the dynamical system that goes through the point in the full space. Hence, the reader should imagine each of these planes crossed by many trajectories as the ones shown here, each one intersecting the plane at a unique point. We then filter the intersection points by selecting those that fulfil the ramp condition. Then we repeat this process for different values of the

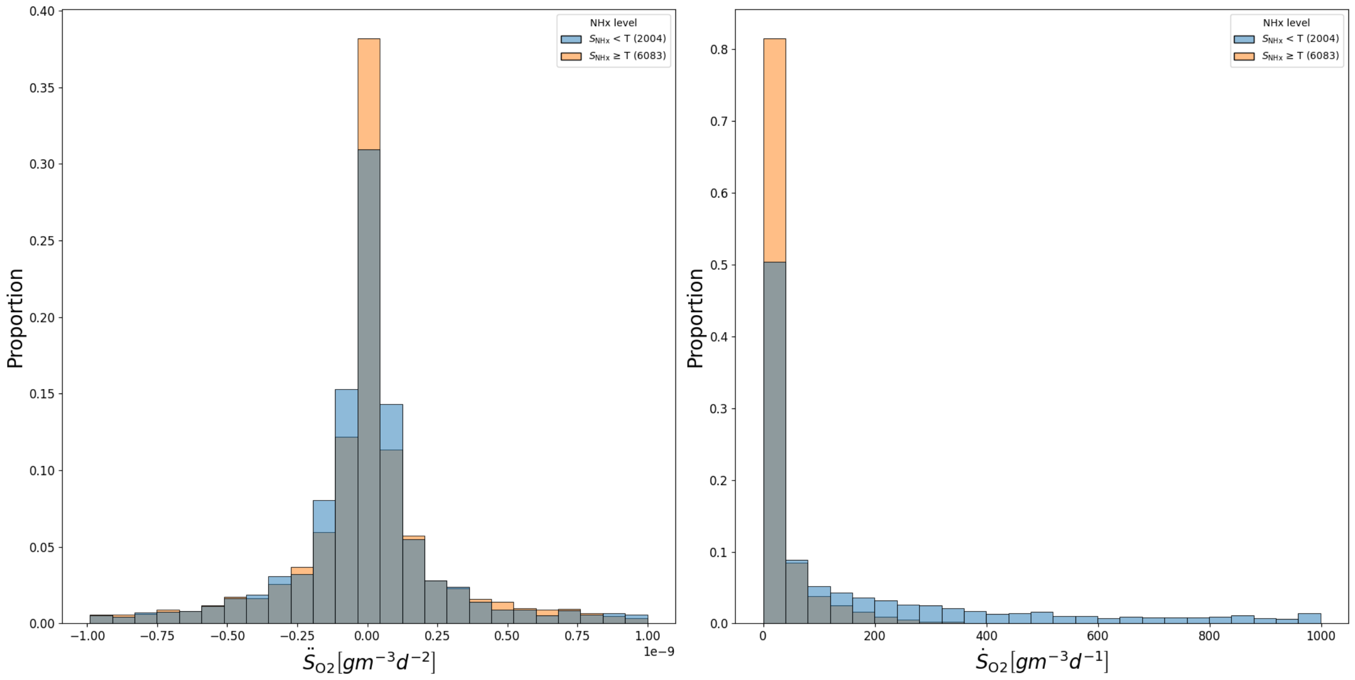

Figure 3: Values of the dissolved oxygen time derivatives of the selected samples from fig.6 in main article

states in $P^{\perp}$ (different planes in the figure). We collect all these points in a single plane. These are the curves shown in fig.5 in the body of the article.

The points shown in fig. 6 are obtained in a similar fashion. However, for these points, we do not fix any state. All states (including S_{NHx}) are free, and to sample the hyper-surface defined by the ramp conditions (9) and (10) in the main article, we solve an optimisation problem, which can be stated as follows: generate distinct points in a level set defined by a function $\phi : \mathbb{R}^n \to \mathbb{R}$ (in our case $n = 8$):

$$\mathscr{L}_c := \{x \in \mathbb{R}^n : \phi(x) = c\} \tag{22}$$

To warrant good behaviour of the set, we assume that the function has the following properties:

1. is continuous: implies closedness of the sets $\{x \in \mathbb{R}^n : \phi(x) \leq c\}$ (sublevel set)
2. $\nabla\phi(x) \neq 0 \quad \forall x \in \mathscr{L}_c$ (non-degenerate level set, level sets do not intersect)
3. is coercive: $\lim_{\|x\|\to\infty} \phi(x) = \infty$ (compact sublevel sets). That is: ϕ is coercive $\iff \forall c \in \mathbb{R}, \{x \in \mathbb{R}^n : \phi(x) \leq c\}$ is compact.

We can set the problem of sampling the ramp hyper-surface as a constrained optimisation:

$$x = \underset{x \in S \subset \mathbb{R}^n}{\arg\min} f(x, \theta) \tag{23}$$
$$\phi(x) = 0 \quad \text{eq. 9 main article} \tag{24}$$
$$\psi(x) > 0 \quad \text{eq. 10 main article} \tag{25}$$

where S is chosen such that $\mathscr{L}_c \cap S \neq \emptyset$ (e.g. a hyperbox), and the cost function $f(x) : \mathbb{R}^n \to \mathbb{R}$ is arbitrary. The quality of the optimisation results for our sampling can be verified by looking at the distributions shown in fig. 3. There, the values of $\phi(x)$ are shown (second time derivative, which should be zero), together with the values of $\psi(x)$ (first time derivative, must be positive).

The cost function is exploited to achieve other objectives, like the spread of the points over the surface. For example:

$$f(x, \{x_i\}_{1\leq i\leq I}, r_{\min}) = -\sum_{i=1}^{I} H(r_{\min} - \|x - x_i\|)\|x - x_i\|^2 \tag{26}$$

where $\{x_i\}_{1\leq i\leq I}$ is a set of points in $\mathscr{L}_c$ (e.g. the set of previous solutions to the problem). $H(r)$ is the step function, with value 1 when $r > 0$.

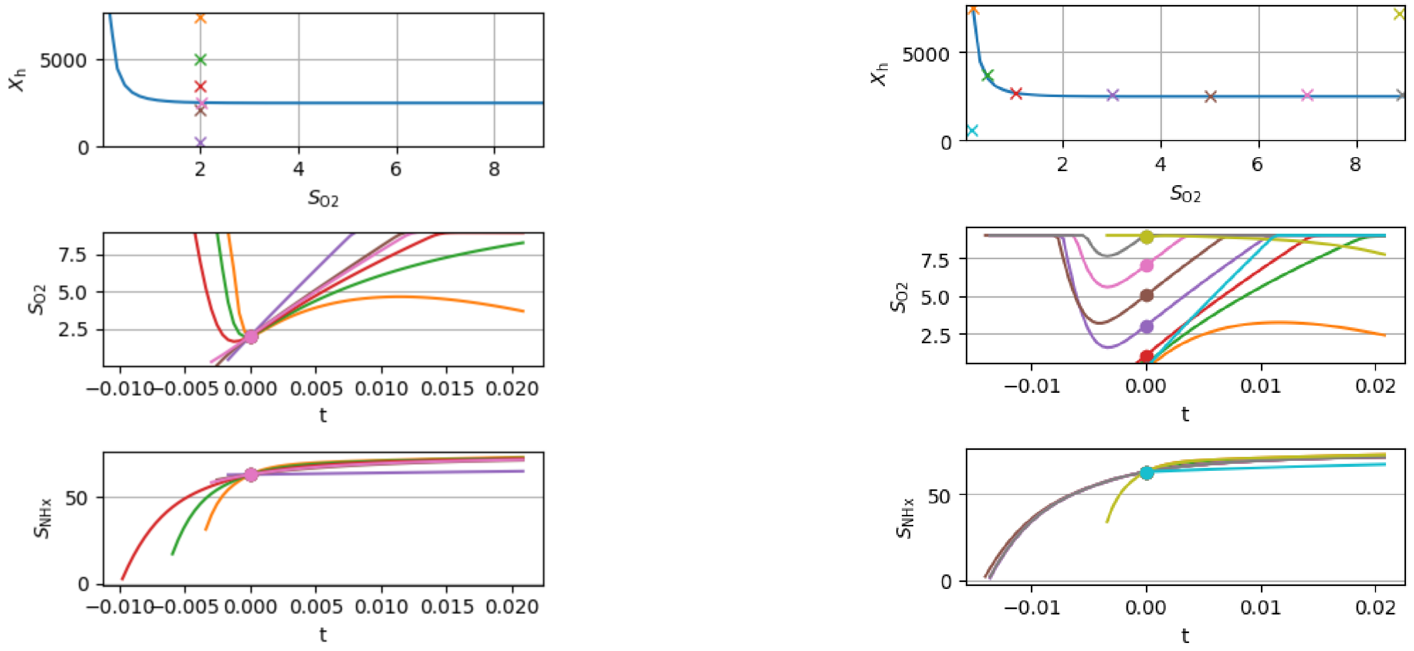

(a) Reconstruction for $S_{\mathrm{O2}} = 2\,\mathrm{g\,m^{-3}}$.

(b) Reconstruction along the inflection point curve.

Figure 4: Reconstruction of the S_{O2} and S_{NHx} curve from selected points, marked with an x, in the S_{O2}, X_{h} plane. In a) only one value is on the inflection point curve, hence has a ramp, in b) all but the two in the bottom left and upper right corner are combinations of states with ammonium above the threshold leading to ramps.

Numerical solutions using Sequential Quadratic Programming (SQP) showed an accumulation of solutions on the boundary of the intersection $\mathcal{L}_c \cap S$, likely due to the Karush–Kuhn–Tucker conditions. Other constrained optimisers are to be tested, but as a workaround, the cost function can be extended to

$$\tilde{f}(x, \{x_i\}_{1\leq i\leq I}, \partial S) = f(x, \{x_i\}_{1\leq i\leq I}) + \mathrm{spike}(x, \partial S) \tag{27}$$

where spike is a function that increases rapidly as x approaches the boundary (∂S) of S. This workaround and a very tight hyperbox provided the reported results.

2.1 Interpretability of the inflection point curve

Figure 4 is the result from an interactive plot where points in the $(X_{\mathrm{h}}, S_{\mathrm{O2}})$ plane can be selected and the time-series are reproduced from these points. The point on the blue line in the top figures are ramps, the points away from the curve or instances where no ramp occurs. From these points then the dissolved oxygen signals are reconstructed. This illustrates the simulation time that we save by computing the inflection point curve directly. Hence, we evaluated ramps for 100,000 initial conditions in a few minutes on a normal laptop, which covers a large region of the state-space.

3 Symbols name

Table 1 introduces the names of the symbols used in this work. Table 2 introduces the naming of the rates.

This work	Other name	Description
S_{b}	S_{B}	Soluble biodegradable organics (SS)
S_{nb}	S_{U}	Soluble nondegradable organics (SI)
S_{O2}	S_{O2}	Dissolved oxygen (SO)
X_{cb}	XC_{B}	Particulate and colloidal biodegradable organics (XS)
$X_{\mathrm{nb,in}}$	X_{UInf}	Particulate nonbiodegradable organics from the influent (XI)
$X_{\mathrm{nb,e}}$	X_{UE}	Particulate nonbiodegradable endogenous products (XP)
S_{NHx}	S_{NHx}	Ammonia (NH4 + NH3) (SNH)
S_{NOx}	S_{NOx}	Nitrate and nitrite (NO3 + NO2) (considered to be NO3 only for stoichiometry, SNO)
X_{cbN}	XC_{BN}	Particulate and colloidal biodegradable organic N (XND)
S_{bN}	S_{BN}	Soluble biodegradable organic N (SND)
X_{h}	X_{OHO}	Ordinary heterotrophic organisms(XBH)
X_{a}	X_{ANO}	Autotrophic nitrifying organisms (NH4+ to NO3-, XBA)
S_{alk}	S_{Alk}	Alkalinity (HCO3-, SALK)
S_{N2}	S_{N2}	Dissolved nitrogen (gas, N2), not in classic ASM1

Table 1: Naming of states in this work in relation to commonly used names, e.g. [2], which is provided in the bracket in the description. The "other name" column is based on [3].

This work	Other name	Description
g_{hO2}	g_{hO2}	Aerobic growth of heterotrophs
g_{hAn}	g_{hAn}	Anoxic growth of heterotrophs
g_{aO2}	g_{aO2}	Aerobic growth of autotrophs
d_{h}	d_{h}	Decay of heterotrophs
d_{a}	d_{a}	Decay of autotrophs
a_{N}	am_{N}	Ammonification of soluble organic nitrogen
h_{o}	ho	Hydrolysis of entrapped organics
h_{oN}	ho_{N}	Hydrolysis of entrapped organic nitrogen

Table 2: Naming of process rates and their description. The "other name" column is following [3].

4 ASM1 model

$$\boldsymbol{x} = \begin{bmatrix} S_{\mathrm{nb}} \\ S_{\mathrm{b}} \\ X_{\mathrm{nb,in}} \\ X_{\mathrm{cb}} \\ X_{\mathrm{h}} \\ X_{\mathrm{a}} \\ X_{\mathrm{nb,e}} \\ S_{\mathrm{O2}} \\ S_{\mathrm{NOx}} \\ S_{\mathrm{NHx}} \\ S_{\mathrm{bN}} \\ X_{\mathrm{cbN}} \\ S_{\mathrm{alk}} \\ S_{\mathrm{N2}} \end{bmatrix} \tag{28}$$

$$\mathtt{M} = \begin{bmatrix} 0 & 0 & 0 & 0 & 0 & 0 & 0 & 0 \\ m_{S_{\mathrm{b}}}\, g_{\mathrm{hO2}} & m_{S_{\mathrm{b}}}\, g_{\mathrm{hAn}} & 0 & 0 & 0 & 0 & 1 & 0 \\ 0 & 0 & 0 & 0 & 0 & 0 & 0 & 0 \\ 0 & 0 & 0 & m_{X_{\mathrm{cb}}}\, d_{\mathrm{h}} & m_{X_{\mathrm{cb}}}\, d_{\mathrm{a}} & 0 & -1 & 0 \\ 1 & 1 & 0 & -1 & 0 & 0 & 0 & 0 \\ 0 & 0 & 1 & 0 & -1 & 0 & 0 & 0 \\ 0 & 0 & 0 & m_{X_{\mathrm{nb,e}}}\, d_{\mathrm{h}} & m_{X_{\mathrm{nb,e}}}\, d_{\mathrm{a}} & 0 & 0 & 0 \\ m_{S_{\mathrm{O2}}}\, g_{\mathrm{hO2}} & 0 & m_{S_{\mathrm{O2}}}\, g_{\mathrm{aO2}} & 0 & 0 & 0 & 0 & 0 \\ 0 & m_{S_{\mathrm{NOx}}}\, g_{\mathrm{hAn}} & m_{S_{\mathrm{NOx}}}\, g_{\mathrm{aO2}} & 0 & 0 & 0 & 0 & 0 \\ m_{S_{\mathrm{NHx}}}\, g_{\mathrm{hO2}} & m_{S_{\mathrm{NHx}}}\, g_{\mathrm{hAn}} & m_{S_{\mathrm{NHx}}}\, g_{\mathrm{aO2}} & 0 & 0 & 1 & 0 & 0 \\ 0 & 0 & 0 & 0 & 0 & -1 & 0 & 1 \\ 0 & 0 & 0 & m_{X_{\mathrm{cbN}}}\, d_{\mathrm{h}} & m_{X_{\mathrm{cbN}}}\, d_{\mathrm{a}} & 0 & 0 & -1 \\ m_{S_{\mathrm{alk}}}\, g_{\mathrm{hO2}} & m_{S_{\mathrm{alk}}}\, g_{\mathrm{hAn}} & m_{S_{\mathrm{alk}}}\, g_{\mathrm{aO2}} & 0 & 0 & m_{S_{\mathrm{alk}}}\, a_{\mathrm{N}} & 0 & 0 \\ 0 & m_{S_{\mathrm{N2}}}\, g_{\mathrm{hAn}} & 0 & 0 & 0 & 0 & 0 & 0 \end{bmatrix} \tag{29}$$

$$m_{S_\mathrm{b}\,g_\mathrm{hO2}} \coloneqq -\frac{1}{\gamma_\mathrm{h}} \tag{30}$$
$$m_{S_\mathrm{b}\,g_\mathrm{hAn}} \coloneqq -\frac{1}{\gamma_\mathrm{h}} \tag{31}$$
$$m_{X_\mathrm{cb}\,d_\mathrm{h}} \coloneqq 1-\zeta_\mathrm{Xnb,l} \tag{32}$$
$$m_{X_\mathrm{cb}\,d_\mathrm{a}} \coloneqq 1-\zeta_\mathrm{Xnb,l} \tag{33}$$
$$m_{X_\mathrm{nb,e}\,d_\mathrm{h}} \coloneqq \zeta_\mathrm{Xnb,l} \tag{34}$$
$$m_{X_\mathrm{nb,e}\,d_\mathrm{a}} \coloneqq \zeta_\mathrm{Xnb,l} \tag{35}$$
$$m_{S_\mathrm{O2}\,g_\mathrm{hO2}} \coloneqq \frac{\gamma_\mathrm{h}-1}{\gamma_\mathrm{h}} \tag{36}$$
$$m_{S_\mathrm{O2}\,g_\mathrm{aO2}} \coloneqq \frac{\gamma_\mathrm{a}+\iota_\mathrm{COD,NO3}}{\gamma_\mathrm{a}} \tag{37}$$
$$m_{S_\mathrm{NOx}\,g_\mathrm{hAn}} \coloneqq \frac{\gamma_\mathrm{h}-1}{\gamma_\mathrm{h}\iota_\mathrm{NO3,N2}} \tag{38}$$
$$m_{S_\mathrm{NOx}\,g_\mathrm{aO2}} \coloneqq \frac{1}{\gamma_\mathrm{a}} \tag{39}$$
$$m_{S_\mathrm{NHx}\,g_\mathrm{hO2}} \coloneqq -\iota_\mathrm{NXb} \tag{40}$$
$$m_{S_\mathrm{NHx}\,g_\mathrm{hAn}} \coloneqq -\iota_\mathrm{NXb} \tag{41}$$
$$m_{S_\mathrm{NHx}\,g_\mathrm{aO2}} \coloneqq -\iota_\mathrm{NXb}-\frac{1}{\gamma_\mathrm{a}} \tag{42}$$
$$m_{X_\mathrm{cbN}\,d_\mathrm{h}} \coloneqq \iota_\mathrm{NXb}-\iota_\mathrm{NXnb}\zeta_\mathrm{Xnb,l} \tag{43}$$
$$m_{X_\mathrm{cbN}\,d_\mathrm{a}} \coloneqq \iota_\mathrm{NXb}-\iota_\mathrm{NXnb}\zeta_\mathrm{Xnb,l} \tag{44}$$
$$m_{S_\mathrm{alk}\,g_\mathrm{hO2}} \coloneqq -\iota_\mathrm{NXb}\iota_\mathrm{cSNHx} \tag{45}$$
$$m_{S_\mathrm{alk}\,g_\mathrm{hAn}} \coloneqq -\iota_\mathrm{NXb}\iota_\mathrm{cSNHx}+\frac{\iota_\mathrm{cSNOx}\left(\gamma_\mathrm{h}-1\right)}{\gamma_\mathrm{h}\iota_\mathrm{NO3,N2}} \tag{46}$$
$$m_{S_\mathrm{alk}\,g_\mathrm{aO2}} \coloneqq \iota_\mathrm{cSNHx}\left(-\iota_\mathrm{NXb}-\frac{1}{\gamma_\mathrm{a}}\right)+\frac{\iota_\mathrm{cSNOx}}{\gamma_\mathrm{a}} \tag{47}$$
$$m_{S_\mathrm{alk}\,a_\mathrm{N}} \coloneqq \iota_\mathrm{cSNHx} \tag{48}$$
$$m_{S_\mathrm{N2}\,g_\mathrm{hAn}} \coloneqq \frac{1-\gamma_\mathrm{h}}{\gamma_\mathrm{h}\iota_\mathrm{NO3,N2}} \tag{49}$$
$$\tag{50}$$

$$\boldsymbol{r} = \begin{bmatrix} g_\mathrm{hO2}(S_\mathrm{NHx}, S_\mathrm{O2}, S_\mathrm{b}, X_\mathrm{h}) \\ g_\mathrm{hAn}(S_\mathrm{NHx}, S_\mathrm{NOx}, S_\mathrm{O2}, S_\mathrm{b}, X_\mathrm{h}) \\ g_\mathrm{aO2}(S_\mathrm{NHx}, S_\mathrm{O2}, X_\mathrm{a}) \\ d_\mathrm{h}(X_\mathrm{h}) \\ d_\mathrm{a}(X_\mathrm{a}) \\ a_\mathrm{N}(S_\mathrm{bN}, X_\mathrm{h}) \\ h_\mathrm{o}(S_\mathrm{NOx}, S_\mathrm{O2}, X_\mathrm{cb}, X_\mathrm{h}) \\ h_\mathrm{oN}(S_\mathrm{NOx}, S_\mathrm{O2}, X_\mathrm{cbN}, X_\mathrm{cb}, X_\mathrm{h}) \end{bmatrix} \tag{51}$$

$$g_{\mathrm{hO2}}(S_{\mathrm{NHx}}, S_{\mathrm{O2}}, S_{\mathrm{b}}, X_{\mathrm{h}}) \coloneqq S_{\mathrm{NHx}} S_{\mathrm{b}} X_{\mathrm{h}} \mu_{\mathrm{max,h}} \frac{S_{\mathrm{O2}}}{(S_{\mathrm{NHx}} + \kappa_{\mathrm{NHx,h}})(S_{\mathrm{O2}} + \kappa_{\mathrm{O2,h}})(S_{\mathrm{b}} + \kappa_{\mathrm{b}})} \tag{52}$$

$$g_{\mathrm{hAn}}(S_{\mathrm{NHx}}, S_{\mathrm{NOx}}, S_{\mathrm{O2}}, S_{\mathrm{b}}, X_{\mathrm{h}}) \coloneqq S_{\mathrm{NHx}} S_{\mathrm{b}} X_{\mathrm{h}} \mu_{\mathrm{max,h}} \frac{S_{\mathrm{NOx}} \eta_{\mathrm{an,h}} \kappa_{\mathrm{O2,h}}}{(S_{\mathrm{NHx}} + \kappa_{\mathrm{NHx,h}})(S_{\mathrm{O2}} + \kappa_{\mathrm{O2,h}})(S_{\mathrm{b}} + \kappa_{\mathrm{b}})(S_{\mathrm{NOx}} + \kappa_{\mathrm{NOx,h}})} \tag{53}$$

$$g_{\mathrm{aO2}}(S_{\mathrm{NHx}}, S_{\mathrm{O2}}, X_{\mathrm{a}}) \coloneqq \frac{S_{\mathrm{NHx}} S_{\mathrm{O2}} X_{\mathrm{a}} \mu_{\mathrm{max,a}}}{(S_{\mathrm{NHx}} + \kappa_{\mathrm{NHx,a}})(S_{\mathrm{O2}} + \kappa_{\mathrm{O2,a}})} \tag{54}$$

$$d_{\mathrm{h}}(X_{\mathrm{h}}) \coloneqq X_{\mathrm{h}} \beta_{\mathrm{h}} \tag{55}$$

$$d_{\mathrm{a}}(X_{\mathrm{a}}) \coloneqq X_{\mathrm{a}} \beta_{\mathrm{a}} \tag{56}$$

$$a_{\mathrm{N}}(S_{\mathrm{bN}}, X_{\mathrm{h}}) \coloneqq S_{\mathrm{bN}} X_{\mathrm{h}} \lambda_{\mathrm{am}} \tag{57}$$

$$h_{\mathrm{o}}(S_{\mathrm{NOx}}, S_{\mathrm{O2}}, X_{\mathrm{cb}}, X_{\mathrm{h}}) \coloneqq X_{\mathrm{cb}} \lambda_{\mathrm{hyd,b}} \frac{X_{\mathrm{h}}}{X_{\mathrm{cb}} + X_{\mathrm{h}} \kappa_{\mathrm{Xhyd}}} \left(\frac{S_{\mathrm{NOx}} \eta_{\mathrm{hyd,an}} \kappa_{\mathrm{O2,h}}}{(S_{\mathrm{NOx}} + \kappa_{\mathrm{NOx,h}})(S_{\mathrm{O2}} + \kappa_{\mathrm{O2,h}})} + \frac{S_{\mathrm{O2}}}{S_{\mathrm{O2}} + \kappa_{\mathrm{O2,h}}} \right) \tag{58}$$

$$h_{\mathrm{oN}}(S_{\mathrm{NOx}}, S_{\mathrm{O2}}, X_{\mathrm{cbN}}, X_{\mathrm{cb}}, X_{\mathrm{h}}) \coloneqq X_{\mathrm{cbN}} \lambda_{\mathrm{hyd,b}} \frac{X_{\mathrm{h}}}{X_{\mathrm{cb}} + X_{\mathrm{h}} \kappa_{\mathrm{Xhyd}}} \left(\frac{S_{\mathrm{NOx}} \eta_{\mathrm{hyd,an}} \kappa_{\mathrm{O2,h}}}{(S_{\mathrm{NOx}} + \kappa_{\mathrm{NOx,h}})(S_{\mathrm{O2}} + \kappa_{\mathrm{O2,h}})} + \frac{S_{\mathrm{O2}}}{S_{\mathrm{O2}} + \kappa_{\mathrm{O2,h}}} \right) \tag{59}$$

$$\tag{60}$$

$$\dot{S}_{\mathrm{b}} \coloneqq g_{\mathrm{hAn}} m_{S_{\mathrm{b}}\, g_{\mathrm{hAn}}} + g_{\mathrm{hO2}} m_{S_{\mathrm{b}}\, g_{\mathrm{hO2}}} + h_{\mathrm{o}} \tag{61}$$

$$\dot{X}_{\mathrm{cb}} \coloneqq d_{\mathrm{a}} m_{X_{\mathrm{cb}}\, d_{\mathrm{a}}} + d_{\mathrm{h}} m_{X_{\mathrm{cb}}\, d_{\mathrm{h}}} - h_{\mathrm{o}} \tag{62}$$

$$\dot{X}_{\mathrm{h}} \coloneqq -d_{\mathrm{h}} + g_{\mathrm{hAn}} + g_{\mathrm{hO2}} \tag{63}$$

$$\dot{X}_{\mathrm{a}} \coloneqq -d_{\mathrm{a}} + g_{\mathrm{aO2}} \tag{64}$$

$$\dot{S}_{\mathrm{O2}} \coloneqq g_{\mathrm{aO2}} m_{S_{\mathrm{O2}}\, g_{\mathrm{aO2}}} + g_{\mathrm{hO2}} m_{S_{\mathrm{O2}}\, g_{\mathrm{hO2}}} \tag{65}$$

$$\dot{S}_{\mathrm{NOx}} \coloneqq g_{\mathrm{aO2}} m_{S_{\mathrm{NOx}}\, g_{\mathrm{aO2}}} + g_{\mathrm{hAn}} m_{S_{\mathrm{NOx}}\, g_{\mathrm{hAn}}} \tag{66}$$

$$\dot{S}_{\mathrm{NHx}} \coloneqq a_{\mathrm{N}} + g_{\mathrm{aO2}} m_{S_{\mathrm{NHx}}\, g_{\mathrm{aO2}}} + g_{\mathrm{hAn}} m_{S_{\mathrm{NHx}}\, g_{\mathrm{hAn}}} + g_{\mathrm{hO2}} m_{S_{\mathrm{NHx}}\, g_{\mathrm{hO2}}} \tag{67}$$

$$\dot{S}_{\mathrm{bN}} \coloneqq -a_{\mathrm{N}} + h_{\mathrm{oN}} \tag{68}$$

$$\dot{X}_{\mathrm{cbN}} \coloneqq d_{\mathrm{a}} m_{X_{\mathrm{cbN}}\, d_{\mathrm{a}}} + d_{\mathrm{h}} m_{X_{\mathrm{cbN}}\, d_{\mathrm{h}}} - h_{\mathrm{oN}} \tag{69}$$

$$\tag{70}$$

$$\dot{X}_{\mathrm{nb,e}} \coloneqq d_{\mathrm{a}} m_{X_{\mathrm{nb,e}}\, d_{\mathrm{a}}} + d_{\mathrm{h}} m_{X_{\mathrm{nb,e}}\, d_{\mathrm{h}}} \tag{71}$$

$$\dot{S}_{\mathrm{alk}} \coloneqq a_{\mathrm{N}} m_{S_{\mathrm{alk}}\, a_{\mathrm{N}}} + g_{\mathrm{aO2}} m_{S_{\mathrm{alk}}\, g_{\mathrm{aO2}}} + g_{\mathrm{hAn}} m_{S_{\mathrm{alk}}\, g_{\mathrm{hAn}}} + g_{\mathrm{hO2}} m_{S_{\mathrm{alk}}\, g_{\mathrm{hO2}}} \tag{72}$$

$$\dot{S}_{\mathrm{N2}} \coloneqq g_{\mathrm{hAn}} m_{S_{\mathrm{N2}}\, g_{\mathrm{hAn}}} \tag{73}$$

$$\tag{74}$$

$$\dot{S}_{\mathrm{nb}} \coloneqq 0 \tag{75}$$

$$\dot{X}_{\mathrm{nb,in}} \coloneqq 0 \tag{76}$$

$$\tag{77}$$

This work	Other name	Value	Units	Description
M_{N}	M_{N}	14.0	$\mathrm{g\,mol^{-1}}$	atomic molar mass of nitrogen
$\mathrm{COD_C}$	COD_{C}	32.0	$\mathrm{g\,mol^{-1}}$	Theoretical COD of molar carbon
$\mathrm{COD_{Fe}}$	COD_{Fe}	24.0	$\mathrm{g\,mol^{-1}}$	Theoretical COD of molar iron
$\mathrm{COD_H}$	COD_{H}	8.0	$\mathrm{g\,mol^{-1}}$	Theoretical COD of molar hydrogen
$\mathrm{COD_N}$	COD_{N}	-24.0	$\mathrm{g\,mol^{-1}}$	Theoretical COD of molar nitrogen
$\mathrm{COD_O}$	COD_{O}	-16.0	$\mathrm{g\,mol^{-1}}$	Theoretical COD of molar oxygen
$\mathrm{COD_P}$	COD_{P}	40.0	$\mathrm{g\,mol^{-1}}$	Theoretical COD of molar phosphorus
$\mathrm{COD_S}$	COD_{S}	48.0	$\mathrm{g\,mol^{-1}}$	Theoretical COD of molar sulphur
$\mathrm{COD_+}$	COD_{pos}	-8.0	$\mathrm{g\,mol^{-1}}$	Theoretical COD of positive charge
$\mathrm{COD_-}$	COD_{neg}	8.0	$\mathrm{g\,mol^{-1}}$	Theoretical COD of negative charge
β_{a}	b_{ANO}	0.15	$\mathrm{d^{-1}}$	Decay rate for XANO (b_{A})
β_{h}	b_{OHO}	0.62	$\mathrm{d^{-1}}$	Decay rate for XOHO (b_{H})
$\eta_{\mathrm{an,h}}$	n_{mOHOAx}	0.8	–	Reduction factor for anoxic growth of XOHO (η_{g})
$\eta_{\mathrm{hyd,an}}$	n_{qhydAx}	0.4	–	Correction factor for hydrolysis under anoxic conditions (η_{h})
γ_{a}	Y_{ANO}	0.24	$\mathrm{g\,g^{-1}}$	Yield of XANO growth per SNO3 (Y_{A})
γ_{h}	Y_{OHO}	0.67	$\mathrm{g\,g^{-1}}$	Yield for XOHO growth (Y_{H})
$\iota_{\mathrm{COD,N2}}$	i_{CODN2}	$\mathrm{COD_N}/M_{\mathrm{N}}$	$\mathrm{g\,g^{-1}}$	Conversion factor for N2 in COD (i_{CODN2})
$\iota_{\mathrm{COD,NO3}}$	i_{CODNO3}	$(\mathrm{COD_N} + 3\mathrm{COD_O} + \mathrm{COD_-})/M_{\mathrm{N}}$	$\mathrm{g\,g^{-1}}$	Conversion factor for NO3 in COD (i_{CODNO3})
$\iota_{\mathrm{NO3,N2}}$	i_{NO3N2}	$(-3\mathrm{COD_O} - \mathrm{COD_-})/M_{\mathrm{N}}$	$\mathrm{g\,g^{-1}}$	Conversion factor for NO3 reduction to N2 (i_{NO3N2})
ι_{NXb}	i_{NXBio}	0.086	$\mathrm{g\,g^{-1}}$	N content of biomass, meaning XOHO, XPAO, and XANO (i_{XB})
ι_{NXnb}	i_{NXUE}	0.06	$\mathrm{g\,g^{-1}}$	N content of products from biomass (i_{XE})
ι_{cSNHx}	$i_{\mathrm{ChargeSNHx}}$	$1/M_{\mathrm{N}}$	$\mathrm{mol\,g^{-1}}$	Conversion factor for NHx in charge ($i_{\mathrm{ChargeSNHx}}$)
ι_{cSNOx}	$i_{\mathrm{ChargeSNOx}}$	$-1/M_{\mathrm{N}}$	$\mathrm{mol\,g^{-1}}$	Conversion factor for NO3 in charge ($i_{\mathrm{ChargeSNOx}}$)
κ_{b}	K_{SBOHO}	20.0	$\mathrm{g\,m^{-3}}$	Half-saturation coefficient for SB (K_{S})
$\kappa_{\mathrm{NHx,a}}$	K_{NHxANO}	1.0	$\mathrm{g\,m^{-3}}$	Half-saturation coefficient for SNHx for XANO (K_{NH})
$\kappa_{\mathrm{NHx,h}}$	K_{NHxOHO}	0.05	$\mathrm{g\,m^{-3}}$	Half-saturation coefficient for NH4 (not in classic ASM1)
$\kappa_{\mathrm{NOx,h}}$	K_{NOxOHO}	0.5	$\mathrm{g\,m^{-3}}$	Half-saturation coefficient for SNOx XOHO (K_{NO})
$\kappa_{\mathrm{O2,a}}$	K_{O2ANO}	0.4	$\mathrm{g\,m^{-3}}$	Half-saturation coefficient for SO2 for XANO (K_{OA})
$\kappa_{\mathrm{O2,h}}$	K_{O2OHO}	0.2	$\mathrm{g\,m^{-3}}$	Half-saturation coefficient for SO2 XOHO (K_{OH})
κ_{Xhyd}	K_{XCBhyd}	0.03	$\mathrm{g\,g^{-1}}$	Saturation coefficient for XB/XOHO (K_{X})
λ_{am}	q_{am}	0.08	$\mathrm{m^3\,d^{-1}\,g^{-1}}$	Rate constant for ammonification (k_{a})
$\lambda_{\mathrm{hyd,b}}$	q_{XCBSBhyd}	3.0	$\mathrm{g\,g^{-1}}$	Maximum specific hydrolysis rate of particulate and soluble biodegradable organics (k_{h})
$\mu_{\mathrm{max,h}}$	m_{OHOMax}	6.0	$\mathrm{d^{-1}}$	Maximum growth rate of XOHO (μ_{H})
$\mu_{\mathrm{max,a}}$	m_{ANOMax}	0.8	$\mathrm{d^{-1}}$	Maximum growth rate of XANO (μ_{A})
$\zeta_{\mathrm{Xnb,l}}$	f_{XUBiolys}	0.08	$\mathrm{g\,g^{-1}}$	Fraction of XU generated in biomass decay (f_{P})

Table 3: Parameters

State variable	min	reference	max	reference
S_{b}	0.9	reactor 5 [4]	69	[4]
S_{O2}	2	[5]	2.2	[5]
X_{cb}	49	reactor 5 [4]	82	reactor 1 [4]
S_{NHx}	1.7	reactor 5 [4]	8	reactor 1 [4]
S_{NOx}	6	reactor 1 [4]	10	reactor 5 [4]
X_{cbN}	0.01	reactor 1 [4]	3.5	reactor 5 [4]
S_{bN}	0.7	reactor 5 [4]	2	reactor 1 [4]
X_{h}	2304	reactor 5 [4]	2816	reactor 1 [4]
X_{a}	135	reactor 5 [4]	165	reactor 1 [4]

Table 4: Scenario 1 for range of concentrations in the reactor that the wastewater treatment process is designed for. Most values are taken from the Benchmark Simulation Model1 (BSM) [4]. The dissolved oxygen S_{O2} concentration is an exception as the model is not for a sequencing batch reactor and we used the values that we observed in an sequencing batch reactor. With this selection method X_{h} and X_{a} have the same value which is not suitable for our approach. Hence we took for the maximum +10% and for the minimum -10% from the value in the BSM1 which are 2560 respectively 150.

State variable	min	reference	max	reference
S_{b}	0	-	2560	[6]
S_{O2}	0	-	8	saturation concentration
X_{cb}	0.2	effluent [4]	91	mean of reactor 1 and underflow [4]
S_{NHx}	0	-	400	assumption based on [7]
S_{NOx}	0	-	400	assumption based on [7]
X_{cbN}	0.01	effluent [4]	6.5	mean of reactor 1 and underflow [4]
S_{bN}	0.7	reference	320	[8]
X_{h}	10	effluent [4]	3780	mean of reactor 1 and underflow [4]
X_{a}	0.6	effluent [4]	225	mean of reactor 1 and underflow [4]

Table 5: Scenario 2 for range of concentrations in the reactor which could happen in for example on-site wastewater treatment facilities. The assumption based on Dockhorn et al. [7] is made that all nitrogen produces per day is present as NHx respectively NOx and that 60 liters of water are consumed per day.

State variable	min	reference	max	reference
S_{b}	0	-	8200	observed maximum[9]
S_{O2}	0	-	8	saturation concentration
X_{cb}	0	-	100	underflow [4]
S_{NHx}	0	-	300000	saturation concentration
S_{NOx}	0	-	9000	fertiliser production [10]
X_{cbN}	0	-	7	underflow [4]
S_{bN}	0	-	320	[8]
X_{h}	0	-	5000	underflow [4]
X_{a}	0	-	300	underflow [4]

Table 6: Scenario 3 for range of concentrations in the reactor which are physically possible, though many probably unlikely to happen.

5 Causal diagram

Confounding situations are when one sees an association (correlation) but there is no causal relationship. A mediator M (X -> M -> F) might be present or the situation is spurious (X <- F -> Y), which means that there is no causal relation that leads to the correlation between the two elements X and Y. Another situation in causal diagrams is a collider (X -> F <- Y), which is also called a V-structure or immorality and is the structure that we observed between the different states of the ASM1 and the ramp feature. The V-structure means that not only the variable of interest X leads to feature F (S_{NHx} < threshold), but also several other state variables such as Y.

6 Ammonium depletion soft-sensor based on the dissolved oxygen ramp feature

In a previous study [5], the soft sensor for a maintained dissolved oxygen sensor reached a 93% accuracy with the ramp feature for a balanced dataset where half of the data had an ammonium concentration below or equal to a threshold T and half the data was above. The arbitrary threshold was chosen as double the amount of the detection limit of the ammonium measurements in the laboratory (i.e. $1\,\mathrm{g\,m^{-3}}$). For the unmaintained sensor, it was 80%. A high prediction accuracy could be achieved despite our study exposing alternative causes for the ramp.

In the previous study, we chose the most challenging real-world cases to which we were granted access. Additionally, we used synthetic, modelled data to make the feature fail to validate the feature and still obtained a wide range with high prediction accuracy [11].

Nevertheless, despite the rigour that we used, the results of the Dynamical Systems Analysis (DSA) in this article clearly advise caution when barely deciding on fit in data-matching if a prediction is good enough or not. Especially in the face of climate change or on-site applications where conditions outside the training data set are likely to occur. This supports the statement by Zhong et al. who show the power of data-driven tools, but also caution that they should not be overly trusted either. DSA hence should indeed be applied much more frequently to decide if a feature is really robust or needs to be further refined or combined with other features, as the ramp feature will have to be.

References

[1] Juan Pablo Carbajal and Mariane Yvonne Schneider. dynamical systems analysis, 2024. URL `https://gitlab.com/sbrml/dsa-signal-features/`.

[2] Mogens Henze, Willi Gujer, Takahashi Mino, and M. C. M. van Loosdrecht. *Activated sludge models ASM1, ASM2, ASM2d and ASM3*. IWA publishing. IWA, Londen, UK, 2000. ISBN 978-1-900222-24-2.

[3] H. Hauduc, L. Rieger, I. Takács, A. Héduit, P. A. Vanrolleghem, and S. Gillot. A systematic approach for model verification: application on seven published activated sludge models. *Water Science and Technology*, 61(4):825–839, February 2010. ISSN 0273-1223. doi: 10.2166/wst.2010.898. _eprint: https://iwaponline.com/wst/article-pdf/61/4/825/1044907/825.pdf.

[4] J. Alex, L. Benedetti, J. Copp, K.V. Gernaey, Ulf Jeppsson, I. Nopens, M.-N. Pons, J.-P. Steyer, and P.A. Vanrolleghem. *Benchmark Simulation Model no. 1 (BSM1)*, volume TEIE-7229. Lund University, 2008.

[5] Mariane Yvonne Schneider, Juan Pablo Carbajal, Viviane Furrer, Bettina Sterkele, Max Maurer, and Kris Villez. Beyond signal quality: The value of unmaintained pH, dissolved oxygen, and oxidation-reduction potential sensors for remote performance monitoring of on-site sequencing batch reactors. *Water Research*, 161:639–651, September 2019. ISSN 00431354. doi: 10.1016/j.watres.2019.06.007.

[6] Patcharin Racho and Apinya Pongampornnara. Enhanced biogas production from modified tapioca starch wastewater. *Energy Reports*, 6:744–750, 2020. ISSN 2352-4847. doi: https://doi.org/10.1016/j.egyr.2019.09.058.

[7] Thomas Dockhorn, Joerg Felmeden, Thomas Hillenbrand, I Kaufmann Alves, Bernd Kirschbaum, Guenter Langergraber, Sabine Lautenschlaeger, Max Maurer, Silke Neuhausen, Julia Sigglow, and Heidrun Steinmetz. *Arbeitsblatt DWA-A 272 Grundsätze für die Planung und Implementierung Neuartiger Sanitärsysteme (NASS)*. DWA-Regelwerk. DWA, Hennef, Germany, first edition, June 2014. ISBN 978-3-944328-63-8.

[8] Seow Wah How, Jia Huey Sin, Sharon Ying Ying Wong, Pek Boon Lim, Alijah Mohd Aris, Gek Cheng Ngoh, Tadashi Shoji, Thomas P. Curtis, and Adeline Seak May Chua. Characterization of slowly-biodegradable organic compounds and hydrolysis kinetics in tropical wastewater for biological nitrogen removal. *Water Science and Technology*, 81(1):71–80, February 2020. ISSN 0273-1223. doi: 10.2166/wst.2020.077.

[9] Kathryn S Lowe, Maria B Tucholke, Jill M.B Tomaras, Kathleen Conn, Christiane Hoppe, Jörg E Drewes, John E McCray, and Junko Munakata-Marr. Influent Constituent Characteristics of the Modern Waste Stream from Single Sources: Final Report, September 2009. URL `http://web.archive.org/web/20190216024758/http://www.decentralizedwater.org/research_project_04-DEC-1.asp`.

[10] Pradip B. Dhamole, Rashmi R. Nair, Stanislaus F. D'Souza, and S. S. Lele. Denitrification of high strength nitrate waste. *Bioresource Technology*, 98(2):247–252, January 2007. ISSN 0960-8524. doi: 10.1016/j.biortech.2006.01.019.

[11] Mariane Yvonne Schneider, Viviane Furrer, Eleonora Sprenger, Juan Pablo Carbajal, Kris Villez, and Max Maurer. Benchmarking Soft Sensors for Remote Monitoring of On-Site Wastewater Treatment Plants. *Environmental Science & Technology*, 54(17):10840–10849, September 2020. ISSN 0013-936X, 1520-5851. doi: 10.1021/acs.est.9b07760.

[12] Shifa Zhong, Kai Zhang, Majid Bagheri, Joel G. Burken, April Gu, Baikun Li, Xingmao Ma, Babetta L. Marrone, Zhiyong Jason Ren, Joshua Schrier, Wei Shi, Haoyue Tan, Tianbao Wang, Xu Wang, Bryan M. Wong, Xusheng Xiao, Xiong Yu, Jun-Jie Zhu, and Huichun Zhang. Machine Learning: New Ideas and Tools in Environmental Science and Engineering. *Environmental Science & Technology*, 55(19):12741–12754, October 2021. ISSN 0013-936X. doi: 10.1021/acs.est.1c01339. Publisher: American Chemical Society.
